# Development, Characterization, and Antimicrobial Evaluation of Hybrid Nanoparticles (HNPs) Based on Phospholipids, Cholesterol, Colistin, and Chitosan Against Multidrug-Resistant Gram-Negative Bacteria

**DOI:** 10.3390/pharmaceutics17020182

**Published:** 2025-02-01

**Authors:** Isabella Perdomo, Carolina Mora, Juan Pinillos, José Oñate-Garzón, Constain H. Salamanca

**Affiliations:** 1 Grupo Natura, Departamento de Ciencias Farmacéuticas y Químicas, Facultad de Ingeniería, Diseño y Ciencias Aplicadas, Universidad Icesi, Calle 18 No. 122-135, Cali 760031, Colombia; bella1999isa@gmail.com (I.P.); cmora@icesi.edu.co (C.M.); jfpinillos@icesi.edu.co (J.P.); 2 Grupo de Investigación en Química y Biotecnología (QUIBIO), Facultad de Ciencias Básicas, Universidad Santiago de Cali, Cali 760035, Colombia; 3 Grupo de Investigación Biopolimer, Departamento de Farmacia, Facultad de Ciencias Farmacéuticas y Alimentarias, Universidad de Antioquia, Calle 67 No. 53-108, Medellín 050010, Colombia; 4 Grupo de Investigación Ciencia de Materiales Avanzados, Departamento de Química, Facultad de Ciencias, Universidad Nacional de Colombia sede Medellín, Cra. 65 #59a-110, Medellín 050034, Colombia

**Keywords:** hybrid nanoparticles, chitosan, colistin, *Klebsiella pneumoniae*, multidrug-resistant strains, phospholipids

## Abstract

**Background:** Colistin, a lipopeptide antibiotic usually used as a last resort against multidrug-resistant bacterial strains, has also begun to address the challenge of antimicrobial resistance. **Objective**: this study evaluates whether hybrid nanoparticles (HNPs) composed of Phospholipon^®^ 90G, cholesterol, and colistin can enhance its effectiveness against resistant clinical isolates of *Klebsiella pneumoniae*, a clinically significant Gram-negative bacterium. **Methods:** HNPs were developed using the ethanol injection method and coated with chitosan through a layer-by-layer technique. HNP characterization included measurements of particle size, polydispersity index (PDI), and zeta potential, along with thermal (DSC) and spectrophotometric (FT-IR) analyses. Ultrafiltration and ATR-FTIR were employed to assess colistin’s association and release efficiencies. The biological evaluation followed CLSI guidelines. **Results:** uncoated hybrid nanoparticles (U-HNP) and chitosan-coated hybrid nanoparticles (Ch-HNP) described monodisperse populations, with respective PDI values of ~0.124 and ~0.150, Z-averages of ~249 nm and ~250 nm, and zeta potential values of +17 mV and +20 mV. Colistin’s association and release efficiencies were approximately 79% and 10%, respectively. Regarding antimicrobial activity, results showed that colistin as part of HNPs is poorly effective against this microorganism. However, in the most resistant strain, colistin activity increased slightly when the HNP was coated with chitosan. **Conclusions:** HNPs described high stability against disaggregation, limiting the colistin release and, therefore, affecting antimicrobial performance.

## 1. Introduction

Antimicrobial resistance represents an escalating global crisis that has been developing over centuries. Since the introduction of antibiotics, bacteria have continually evolved mechanisms to resist their effects. The misuse and overuse of antibiotics have accelerated the rise in resistant bacterial strains, leading to a global health crisis with many antibiotics becoming ineffective [1,2,3].

Colistin, also known as polymyxin E, has re-emerged as a critical last-resort antibiotic for treating infections caused by multidrug-resistant (MDR) Gram-negative bacteria [4]. As a cationic polypeptide, colistin targets the outer membrane of Gram-negative bacteria, disrupting lipid membrane integrity, which is essential for bacterial survival and function [5,6]. However, the reintroduction of polymyxins, particularly colistin, has inadvertently created selective pressure, contributing to the emergence of “superbugs”. These resistant strains, including *Pseudomonas aeruginosa*, *Acinetobacter baumannii*, and *Klebsiella pneumoniae*, have been increasingly reported worldwide [7,8,9,10,11,12].

One of the most challenging pathogens corresponding to *Klebsiella pneumoniae* is a Gram-negative bacterium belonging to the Enterobacteriaceae family, which can cause several serious nosocomial infections with high mortality rates, such as pneumonia, urinary tract infections, sepsis, soft tissue infections, and surgical wound infections. This microorganism has been describing serious health problems due to the generation of antibiotic resistance through carbapenemases, which are becoming increasingly resistant [7,13]. The rise in these antibiotic-resistant bacterial strains has elevated its threat level, placing it on the critical priority list of pathogens that pose the most significant risk to human health [14]. MDR isolates of *Klebsiella pneumoniae* carry resistance genes against various antibiotics, including beta-lactams, carbapenems, aminoglycosides, fluoroquinolones, and polymyxins [15,16]. Polymyxin resistance is often mediated by modifications to lipopolysaccharides (LPS), such as changes in the O-antigen length, lipid A modifications with phosphoethanolamine (PEtN) and 4-amino-4-deoxy-L-arabinose (L-Ara4N), and deacylation [17].

The gap between novel antibiotic discovery and the rise in resistant bacterial strains underscores the urgent need for new strategies, with nano-systems like liposomes presenting a promising solution to this challenge [18]. Nanoliposomes are spherical vesicles with excellent biocompatibility that can encapsulate or associate with both hydrophilic and hydrophobic bioactive compounds, protecting them from chemical degradation and facilitating their targeted release [19]. Liposomes have become one of the most promising drug delivery systems in recent years due to their unique properties. Research shows that encapsulating antibiotics within liposomes can enhance their antimicrobial effectiveness. For instance, nanoliposome formulations of colistin have shown promise in treating extracellular bacterial lung infections caused by multidrug-resistant *Pseudomonas aeruginosa*, yielding better results than standard colistin solutions [20,21]. Li et al. (2016) evaluated colistin-entrapped liposomes, which were driven by electrostatic interactions. They found similar minimum inhibitory concentrations (MICs) but a reduced bacterial killing rate compared to the colistin solution [22]. Ichim (2019) demonstrated that colistin-loaded surface-unmodified liposomes were more effective than unencapsulated colistin in treating severe multidrug-resistant infections [23]. The characteristics and behavior of this type of vesicular system can be further optimized by incorporating polymers on their surface. This polymeric coating can enhance bioactivity and improve physicochemical stability [24], drug delivery selectivity [25], and blood circulation [26].

Chitosan is a biocompatible and non-toxic polymer that has demonstrated potential in enhancing the antibacterial activity of certain antimicrobial drugs [27,28,29] and enhancing the physicochemical stability, cellular uptake, and bioavailability of several active ingredients [30]. Pu et al. (2016) discovered that liposomes coated with chitosan, encapsulating the Apep10 peptide, exhibited significantly enhanced stability compared to uncoated liposomes. This finding provides reassurance about the reliability of the research, as the surface coating was found to effectively reduce aggregation [31]. Quin et al. investigated the co-delivery of curcumin and colistin using negatively charged poly (ethylene glycol)-functionalized liposomes, demonstrating potent antibacterial activity against colistin-resistant bacteria. This reassurance about the reliability of the research is further supported by the effectiveness of this innovative drug delivery method compared to the free drug combination [32]. Similarly, Da Silva et al. (2014) evaluated nisin encapsulated in phosphatidylcholine liposomes coated with chitosan and chondroitin sulfate. Their findings highlighted the stability and efficiency of the chitosan-coated formulation in inhibiting *L. monocytogenes*, further reinforcing the reliability of the research [33].

It is important to highlight that currently there are still few studies of the colistin peptide with different types of materials and especially those with vesicular nanostructures and even more so in hybrid nanoparticle systems, which are systems that combine two or more types of nanocomponents [34,35,36,37]. Thus, this study is focused on evaluating a new type of nanostructured system, which has not been reported to date. Such nanostructured system consists of two different types of structures corresponding to phospholipid-cholesterol lamellae and colistin micelle-type aggregates, which in turn were surface coated with the cationic biopolymer chitosan. These new nanostructured systems were optimized considering conventional quality attributes for such systems (particle size ~200 nm, polydispersity < 0.3, zeta potential > 20 mV, and colistin association efficiency >60%). Likewise, its antibiotic activity against multi-resistant clinical isolates of *Klebsiella pneumoniae* was evaluated, offering a hopeful perspective for the future of research in the field of nanoparticles as novel pharmaceutical formulation strategies.

## 2. Materials and Methods

### 2.1. Bacterial Strains and Materials

*K. pneumoniae* MDR strains (Kp-01 and Kp-02) were provided by a Public Health Services Hospital in Valle del Cauca, Colombia. Muller Hinton Cation-Adjusted Broth and Brain Heart Infusion were purchased from Merck (Darmstadt, Hesse, Germany). Phospholipon^®^ 90G was purchased from Lipoid GmbH (Ludwigshafen am Rhein, Rhineland-Palatinate, Germany). This phospholipid material consists of phosphatidylcholine (≥94%), lysophosphatidylcholine (≤4%), α-tocopherol (≤0.3%), and ascorbyl palmitate (≤0.1%) according to the manufacturer’s technical sheet. Chitosan with a high molecular weight (310–375 kDa) and a deacetylation degree over 75%, cholesterol, ethanol, and colistin sulfate were obtained from Sigma Aldrich (St. Louis, MO, USA). Ultrapure water, supplied from an Elix Essential Millipore^®^ purification system, with a mean conductivity value of ~1 µS/cm, was used to maintain the purity of the reagents and the consistency of experimental conditions.

### 2.2. Development and Physicochemical Characterization of Hybrid Nanoparticles (HNPs)

#### 2.2.1. Characterization of the Intrinsic Aggregation of HNPs Materials

The surface behavior of aqueous dispersions of Phospholipon^®^ 90G, chitosan polymer, and colistin peptide was investigated using the pendant drop method. A tensiometer equipped with an optical system for contour analysis (OCA 15EC^®^, Dataphysics Instruments GmbH, Filderstadt, Baden-Württemberg, Germany) and SNP 165/119 needle systems was employed for this study. The analysis was conducted at a temperature of 25 °C ± 0.5 °C and a relative humidity of 65% ± 1%. The collected data were reported in millinewtons per meter (mN/m). Changes in surface tension were measured at various concentration ranges: 4.1 mM to 27.2 mM for Phospholipon^®^ 90G, 0.001 mM to 0.1 mM for chitosan polymer, and 0.07 mM to 23.7 mM for colistin peptide.

#### 2.2.2. Development and Optimization of Uncoated Hybrid Nanoparticles (U-HNPs)

U-HNPs were prepared using the ethanol injection method [38], which has been previously standardized and reported by our laboratory. For this process, the organic and aqueous phases were prepared separately. A total of 5.0 mL of the organic phase was injected dropwise into 5.0 mL of the aqueous phase using a 0.8 mm needle and a controlled flow rate of 0.5 mL/min. The organic phase contained two separate ethanolic dispersions of Phospholipon^®^ 90G at concentrations of 1.5 mg/mL and 3.0 mg/mL, which were then mixed with two ethanolic cholesterol dispersions at concentrations of 0.23 mg/mL and 0.45 mg/mL. In contrast, the aqueous phase consisted of colistin solutions at two concentrations—5.0 mg/mL and 10.0 mg/mL—prepared with sterile, ultrapure water. Once the organic phase was injected into the aqueous phase, the mixture was stirred at 400 RPM for 1 h. Following this, the system was ultrafiltered using an Amicon^®^ Stirred Cell with a capacity of 50 mL, equipped with an Ultracel^®^ ultrafiltration membrane (Darmstadt, Hesse, Germany), which has a cut-off size of 30 kDa. The ultrafiltration was performed at 200 rpm and a nitrogen pressure of 40 psi.

#### 2.2.3. Coating of U-HNPs with Chitosan Polymer

**Figure 1:**
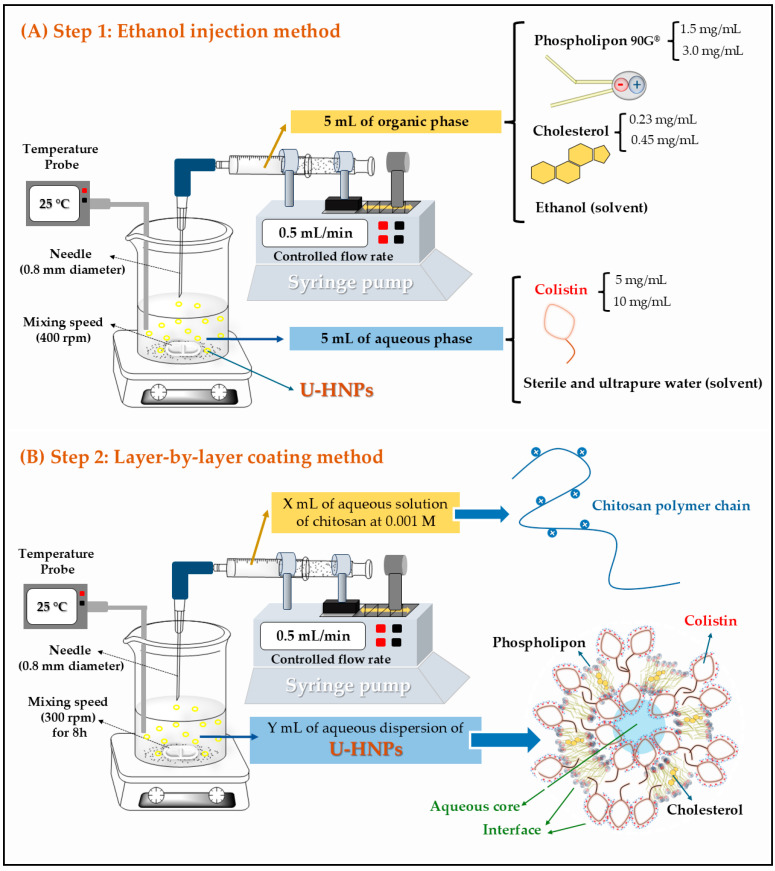
Illustrative diagram of the elaboration process of Hybrid Nanoparticles (HNPs) using two methodologies. (**A**) Ethanol injection method. (**B**) Layer-by-layer coating method on Hybrid Nanoparticle (HNPs). U-HNPs: Uncoated hybrid nanoparticles.

The surface of U-HNPs was modified using the layer-by-layer method [39]. In this process, 7 mL of a 0.001 M aqueous solution of chitosan was added dropwise to 7 mL of an aqueous dispersion containing colistin-formed HNPs, maintaining a 1:1 ratio. The resulting mixture was then stirred continuously at 300 rpm for eight hours in a closed vessel. Figure 1 illustrates the formation process of HNPs through the ethanol injection method, followed by the surface modification using the layer-by-layer coating method.

#### 2.2.4. Characterization of Particle Size, PDI, and Zeta Potential of HNPs

The particle size, polydispersity index (PDI), and zeta potential were measured using a Zetasizer Nano ZSP (Malvern Instruments, Malvern, UK) equipped with a red He/Ne laser operating at 633 nm. Particle size and PDI were determined through dynamic light scattering (DLS) [40] at a scattering angle of 173° and a temperature of 20 °C, using a quartz flow cell (designated as ZEN0023). Zeta potential was measured using a capillary cell (designated as DTS1070). DLS assesses the diffusion of particles under Brownian motion, utilizing a correlation function that follows to the Stokes–Einstein relationship. This method allows for the calculation of particle size and size distribution parameters. Cumulative data form this correlation function, which can be fitted using a simple exponential model to derive the mean size (z-average diameter) and estimate distribution width (PDI). Additionally, the correlation function can be fitted with a multiple exponential model, from which the particle size distribution is obtained using non-negative least squares (NNLS) or constrained regularization (CONTIN). In this study, we referred to the particle size as the z-average diameter. The PDI ranges from 0 to 1, indicating a transition from a monodisperse to a broad-size distribution. For samples prepared for DLS analysis to determine particle size and polydispersity, a dilution factor of 1:10 was used. The same dilution factor was applied for zeta potential measurements, conducted in an aqueous solution of 0.035 mM NaCl.

#### 2.2.5. Characterization of HNPs by DSC and FTIR

To provide additional information on the possible intermolecular interactions among the different components of the HNPs, we conducted a characterization using FTIR and DSC for Phospholipon^®^ 90G, colistin, and chitosan, along with their respective binary mixtures. For this purpose, the binary mixtures were prepared in the exact proportions used in the optimized formulation. Each of these mixtures was dispersed in water, except for Phospholipon^®^ 90G, which was mixed in a hydroalcoholic solution, as outlined in the ethanol injection method. Following this, we removed the aqueous dispersion medium from each mixture through rotary evaporation and freeze-drying processes to obtain a powdery material, which was then characterized using infrared spectrophotometry (FT-IR) and differential scanning calorimetry (DSC). The FT-IR studies were conducted using a Nicolet 6700 Spectrophotometer (ThermoScientific, Waltham, MA, USA), with spectra obtained within the frequency range of 4000 to 400 cm^−^^1^. For the thermal studies, we utilized a Q2000 differential scanning calorimeter (DSC; TA Instruments, New Castle, DE, USA), calibrated with indium (Tm = 155.78 °C, ΔHm = 28.71 J/g). The analysis involved three modulated heating–cooling cycles, ranging from 25 °C (298.15 K) to 400 °C (673.15 K) at a heating rate of 5.0 °C/min, using high-purity nitrogen purge gas (grade 5.0) at a flow rate of 50 mL/min.

#### 2.2.6. HNP Morphological Characterization by TEM

The morphological characterization of chitosan-coated hybrid nanoparticles (Ch-HNPs) was performed using a transmission electron microscope (TEM) JEM-1400 Flash (JEOL Ltd., Akishima, Tokyo, Japan) combined with the negative staining method. To prepare the samples, a drop of each liposomal system was placed on a carbon-coated copper grid and allowed to rest for 1 min. Following this, a drop of 1.0% *w*/*v* uranyl acetate stain was added to the grid. The sample was then allowed to dry at 25 °C for 24 h. Finally, micrographs were obtained at an acceleration voltage of 80 kV.

#### 2.2.7. Association and Release Efficiency of Colistin

An analytical method for quantifying colistin was developed using Attenuated Total Reflectance Fourier Transform Infrared Spectroscopy (ATR-FTIR) with a Nicolet iS10 FTIR Spectrophotometer (ThermoScientific, Waltham, MA, USA). Five spectra of aqueous colistin dispersions at concentrations of 5.0, 15.0, 30.0, 45.0, and 60.0 mg/mL were collected, employing 32 scans in the absorbance range from 2000 to 650 cm^−^^1^, with a spectral resolution of 0.5 cm^−^^1^. The calibration curve was created using the transmittance values of the amide group bending band obtained in the range of 1480–1580 cm^−^^1^ (Y = 2025X + 10,237, R^2^ = 0.9889). The spectra were processed using Omnic 9 software, version 9.8.286 (ThermoScientific, Waltham, MA, USA). To determine the association efficiency (AE) of colistin, ultrafiltration was performed using an Amicon^®^ Stirred Cell. The amount of ultrafiltered colistin was measured and compared against the previously developed calibration curve from ATR-FTIR. Consequently, the encapsulation efficiency (EE) was calculated using the following equation:

(1) $$AE\;\left(\%\right)=\frac{Q_{T}-Q_{UC}}{Q_{T}}\times 100$$

where $Q_{T}$ is the total amount of colistin used in the preparation of HNPs, while $Q_{UC}$ is the amount of ultrafiltered colistin.

In the case of colistin release efficiency (RE), this was determined by preparing a fresh sample of the aqueous dispersions of the HNPs, which were placed in a closed system with constant agitation at 400 rpm ± 5 rpm and at 25 °C ± 1 °C for 16 h. Then, the HNPs were placed in an Amicon^®^ Stirred Cell, where the amount of ultrafiltered colistin was determined and compared against the calibration curve previously developed by ATR-FTIR as previously described. Thus, the colistin release efficiency (RE) was calculated according to the following equation:

(2) $$RE\;(\%)=\frac{Q_{T}-Q_{UC}}{Q_{T}}\times 100$$

where $Q_{T}$ is the total amount of colistin used in the preparation of nanoliposomal systems, while $Q_{UC}$ is the amount of ultrafiltered colistin.

#### 2.2.8. Stability Assay of HNPs

The physical stability of HNPs was investigated using a stress stability assay, which assessed changes in particle size, polydispersity index (PDI), and zeta potential, as outlined in Section 2.2.4. For this purpose, the aqueous dispersions of HNPs were placed in Falcon^®^ 15 mL conical centrifuge tubes and stored in a stability chamber (GAE Ltd., Bogotá, Colombia) at a temperature of 4 °C ± 0.5 °C for one month.

### 2.3. Biological Evaluation

#### 2.3.1. Isolation of Resistant *K. pneumoniae* and Phenotypic Characterization

This study evaluated two clinical isolates of XDR *K. pneumoniae*, designated as Kp-01 and Kp-02. We assessed antibiotic susceptibility, phenotypic characterization of resistance, and determined the minimal inhibitory concentration (MIC) for each antibiotic using the VITEK 2 Antimicrobial Susceptibility Testing card (VITEK N272-AST) in the VITEK system 2, following clinical cut-off points established by the Clinical Laboratory Standards Institute (CLSI) [41]. The antimicrobial agents evaluated in the phenotypic characterization included Piperacillin/Tazobactam (TZP), Ceftazidime (CAZ), Cefepime (FEP), Doripenem (DOR), Imipenem (IPM), Meropenem (MEM), Amikacin (AMK), Gentamicin (GEN), Ciprofloxacin (CIP), and Colistin (CST). Resistance to colistin (CST) was confirmed by conducting a broth microdilution test in glass tubes, with the in vitro MIC determined according to the clinical breakpoints specified by the CLSI [41].

#### 2.3.2. Antimicrobial Activity

Bacterial sensitivity tests were conducted in accordance with the guidelines set by the Clinical and Laboratory Standards Institute [17]. In summary, an overnight culture of *Klebsiella pneumoniae* suspended in Brain Heart Infusion (BHI) broth was diluted with a saline solution (0.45% *v*/*v*) to achieve an optical density of 0.5 on the McFarland scale (approximately 1 × 10^8^ CFU/mL). Following this, an additional dilution factor of 1/150 was applied to reach a concentration of approximately 1 × 10^6^ CFU/mL. One milliliter of this bacterial suspension was then mixed with 10 mL of free colistin and Ch-HNPs, each adjusted to an initial concentration of 128 µg/mL. Serial dilutions were performed to achieve a final concentration of 0.5 µg/mL. The samples were incubated at 37.0 °C ± 0.1 °C for 20 h. This serial dilution process allowed for the determination of the minimum inhibitory concentration (MIC), which is defined as the lowest concentration of the antimicrobial agent at which growth inhibition is observed.

### 2.4. Statistical Analysis

To optimize the HNPs, a 2^K^ (k = 3) factorial design was employed to evaluate the effects of three factors: (i) the concentration of Phospholipon 90G^®^ (1.5 mg/mL and 3.0 mg/mL), (ii) the percentage of cholesterol in the organic phase (0.23%, 0.45%, and 0.90% *w*/*w*), and (iii) the concentration of colistin in the aqueous phase (5.0 mg/mL and 10.0 mg/mL) on the particle size and association efficiency of the HNPs. Atypical data points were identified to assess whether they represented mean or extreme values, and the assumptions of normality and homogeneity of variance were validated. When these assumptions were satisfied, a parametric analysis was conducted using ANOVA, and means were compared using Tukey’s test (α = 0.05). Data analyses were performed with OriginPro version 2023 (Northampton, MA, USA) and GraphPad Prism version 8.0.2 (Northampton, MA, USA) software.

## 3. Results and Discussion

### 3.1. Development and Physicochemical Characterization of Hybrid Nanoparticles (HNPs)

#### 3.1.1. Characterization of the Intrinsic Aggregation of HNPs Materials

**Figure 2:**
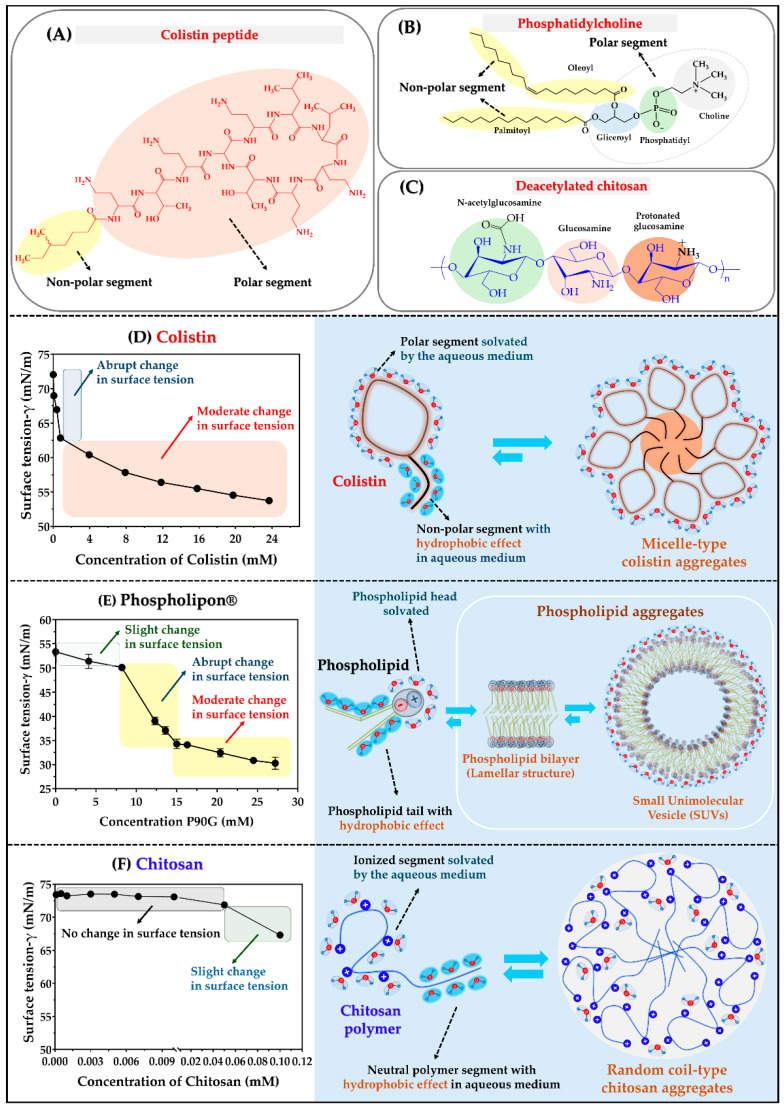
Description of amphiphilic characteristics according to the chemical structures. (**A**) Colistin peptide. (**B**) Phosphatidylcholine (main compound in Phospholipon^®^). (**C**) Chitosan polymer. (**D**) Change in γ regarding Colistin concentration. (**E**) Change in γ regarding Phospholipon^®^ concentration. (**F**) Change in γ regarding Chitosan concentration.

The surface behavior of colistin, Phospholipon^®^ 90G, and chitosan aqueous dispersions is illustrated in Figure 2. Colistin, an amphiphilic peptide (Figure 2A), consists of two segments: a polar head formed by alkyl-amino, hydroxyl, and amide groups, and a non-polar segment made up of a hydrocarbon chain containing six carbon atoms [42]. Likewise, Phospholipon^®^ 90G, composed mainly of phosphatidylcholine, is also an amphiphilic molecule composed of a polar segment (glyceryl-phosphatidyl-choline) and a non-polar segment (two alkyl chains) (Figure 2B) [43]. In contrast, chitosan is a biopolymer derived from chitin [44] and mainly consists of D-glucosamine and N-acetyl-D-glucosamine units (Figure 2C). The N-acetyl-D-glucosamine unit can be deacetylated, resulting in an increased number of glucosamine units that can be protonated in acidic conditions, thus, forming a cationic polyelectrolyte. Consequently, chitosan can achieve a mild to moderate amphiphilic character depending on its degree of deacetylation and the presence of amino and hydroxyl groups in its backbone structure.

Regarding surface tension behavior, colistin demonstrated a significant surfactant effect (Figure 2D), with surface tension decreasing from approximately 72 mN/m to about 54 mN/m. This change occurs in two stages: an initial abrupt drop from ~72 mN/m to ~62 mN/m at low colistin concentrations (0.8 mM), followed by a more gradual decrease from ~62 mN/m to ~54 mN/m between 0.8 mM and 26.7 mM. The first stage can be explained by the rapid migration of colistin to the surface, disrupting the cohesiveness of the aqueous medium (resulting in the abrupt change in surface tension). The second stage can be characterized by a slow and controlled migration, leading to the establishment of a colistin monolayer at the surface (noted by the moderate and consistent change in surface tension). Additionally, colistin at the surface can also move back into the bulk of the aqueous medium, where hydrophobic effects promote a shift in thermodynamic equilibrium towards aggregation, thereby triggering self-assembly in a micellar-like manner [22,45].

In the case of Phospholipon^®^ 90G, a behavior like that of colistin was observed, although with some differences. The surface tension varied from approximately 53 mN/m at 0 mM (in the pure dispersion medium) to about 30.28 mN/m at 27 mM (in concentrated phospholipid dispersion). This change represents a decrease of nearly 57% in the surface tension of the pure hydroalcoholic solution. Additionally, the surface tension profile in relation to phospholipid concentration exhibited typical behavior. A slight change in surface tension was noted from 0 mM to approximately 8 mM, which was followed by a sharp change between 8 mM and 15 mM and then a more moderate change between 15 mM and 26 mM (see Figure 2E). These variations can be explained by several factors. For instance, at a concentration of 0 mM, the maximum surface tension recorded was 53.34 ± 0.07 mN/m, resulting from the cohesiveness of the molecules present in the 7% *V*/*V* ethanolic solution that serves as the dispersion medium. Moreover, Phospholipon^®^ 90G demonstrated an aggregation process, where initially, there was surface saturation. This was followed by a migration of molecules into the bulk of the dispersion medium, leading to a thermodynamic equilibrium that favored vesicular-type self-assembly [46]. Chitosan with a high degree of deacetylation exhibited only a slight change, as seen in Figure 2F. This behavior is expected because chitosan is not an amphiphilic polymer; it tends to be highly soluble in aqueous media rather than at the surface. However, it is important to highlight that this polymer can demonstrate significant interfacial activity, primarily due to the multiple ionic interactions—such as electrostatic and ion–dipole forces—that can occur. Additionally, chitosan can form various types of aggregation structures, ranging from ordered floc-type aggregates to random coil-type aggregates, which arise from the various neutral interactions along its polymeric backbone [47].

#### 3.1.2. Development and Optimization of HNPs

Results of 2^k^ (k = 3) factorial design for the optimization of HNPs are presented in Table 1.

According to the results of the experimental design, run 4 was determined to be the optimal condition for achieving the desired quality characteristics of uncoated hybrid nanoparticles (U-HNPs). This run resulted in a particle size of approximately 200 nm and a polydispersity index (PDI) of less than 0.3. These values indicate the formation of a system with a tendency to produce populations of uniformly sized particles and a high colistin association efficiency exceeding 60%. In this process, U-HNPs were developed using a concentration of 10 mg/mL (~5.7 mM) of colistin in the aqueous phase, along with 1.5 mg/mL (~2.0 mM) of Phospholipon^®^ and 0.23 mg/mL (~0.6 mM) of cholesterol in the organic phase during the ethanol injection method. Consequently, these U-HNPs are primarily composed of colistin, which is approximately three times more concentrated than the phospholipids. This concentration ratio suggests that the developed U-HNPs are formed and interconnected by micelle-type Colistin aggregates, as well as phospholipid lamellar fragments, with smaller amounts of cholesterol incorporated to provide rigidity to the developed nanostructures, as illustrated in Figure 1B.

#### 3.1.3. Characterization of Particle Size, PDI and Zeta Potential of HNPs

**Figure 3:**
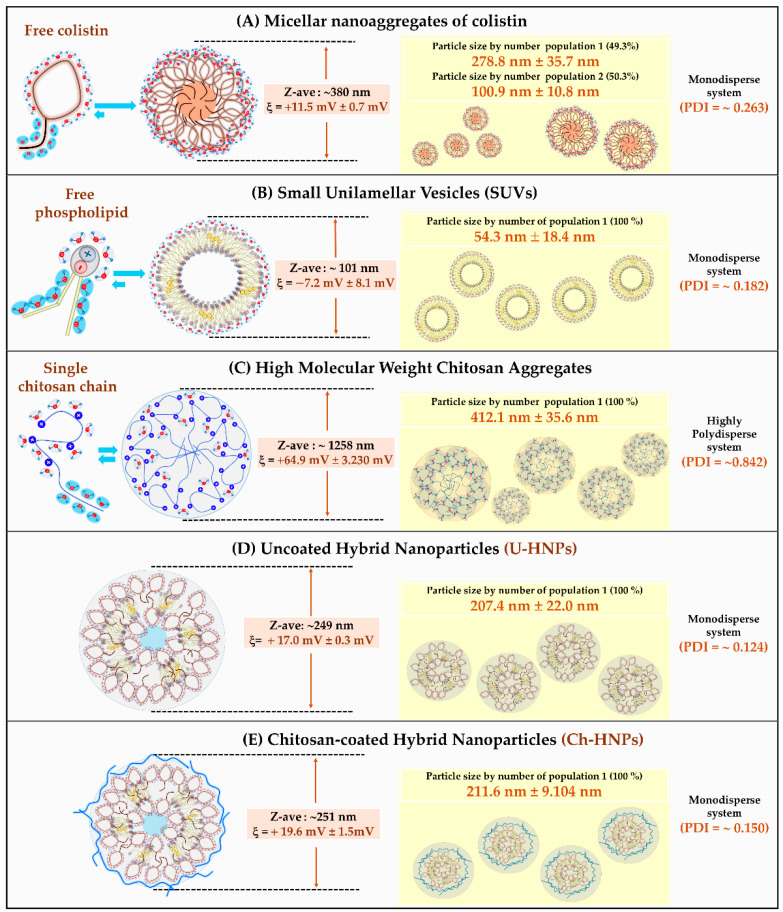
Schematic representation of different aggregation systems, their respective values of Z-average particle size (Z-ave), zeta potential (ξ), particle size by number, and polydispersity index-PDI. (**A**) Micellar nanoaggregates of colistin. (**B**) Small unilamellar vesicles of Phospholipon^®^ 90G. (**C**) High molecular weight of chitosan aggregates. (**D**) Hybrid nanoparticles formed by Phospholipon^®^ 90G, cholesterol, and colistin (U-HNPs). (**E**) Hybrid nanoparticles formed by Phospholipon^®^ 90G, cholesterol, and colistin, coated with high molecular weight chitosan (Ch-HNPs). Note: All recorded data on particle size and zeta potential, obtained through light scattering and capillary electrophoresis, are provided in the Supplementary Materials to ensure the quality of each measurement.

As shown in Figure 3, particle size, polydispersity index (PDI), and zeta potential had different values depending on the intrinsic characteristics of the components and the processes used in the development of HNPs.

In the case of Colistin (Figure 3A), there was a notable tendency towards micellar self-assembly. It was found to have a Z-average particle size of approximately 380 nm, along with two distinct populations: one with a particle size of 278.8 nm ± 35.7 nm and another with a size of 100.9 nm ± 10.8 nm. Additionally, these micellar aggregates exhibited a polydispersity index (PDI) value of about 0.263, indicating low polydispersity in this bimodal system. The Colistin micelles also displayed a slight zeta potential value of +11.5 mV ± 0.7 mV, which is attributed to the interfacial polarization resulting from the protonation of alkyl-amino groups in their polar segments. In the case of Phospholipon^®^ and its primary phospholipid, phosphatidylcholine (Figure 3B), there was a tendency to form lamellar structures that self-assemble into vesicular systems, achieving a Z-average particle size of around 101 nm. These aggregates were found to consist of a single population, with a particle size of 54.3 nm ± 18.4 nm, indicating a monodisperse system (PDI = ~0.182). The particle size distribution and low polydispersity are characteristic of lamellar aggregates and small unilamellar vesicular systems (SUVs) [48]. It was observed that the vesicular aggregates exhibited a slight zeta potential value of +7.2 mV ± 8.1 mV. This value is attributed to the interfacial polarization generated by the ionization of phosphate groups present in the polar segment of phosphatidylcholine. In the case of chitosan (Figure 3C), this polymer tended to form polymeric aggregates with a Z-average particle size of approximately 1258 nm. Additionally, these aggregates presented a single particle size of 412.1 nm ± 35.6 nm, indicating a highly heterodisperse system (PDI = ~0.842), which is consistent with the characteristics of random coil-type aggregates. Furthermore, these polymeric aggregates demonstrated a high zeta potential value of +64.9 mV ± 3.230 mV, attributed to the interfacial polarization from the ionization of D-glucosamine groups. Regarding the uncoated hybrid nanoparticles (U-HNPs) prepared by the ethanol injection method (Figure 3D), the Z-average particle size was found to be approximately 249 nm. These aggregates corresponded to a single-size population of 207.4 nm ± 22.0 nm, with a polydispersity value of ~0.124 and a moderately positive zeta potential of +17.0 mV ± 0.3 mV. These findings support the idea that the development of HNPs is due to the combination of colistin aggregates and Phospholipon^®^ in a highly ordered fashion, leading to the formation of a monodisperse system with a single-size population. The results also indicated that these systems have a slightly positively charged interface, suggesting that the components within the U-HNPs are organized, with the protonated polar heads of colistin primarily oriented toward the interfacial zones. In contrast, the negatively polarized phosphate groups of the phospholipid lamellar aggregates remain inside the nanoparticle, interacting with the polar heads of colistin. This significance of these findings is underscored when comparing the zeta potential values of the individual aqueous dispersions of Phospholipon^®^ (+30.9 mV ± 1 mV) and Colistin (+11.5 mV ± 0.7 mV) to those of the U-HNPs (+17.0 mV ± 0.3 mV), where a positive zeta potential value prevails. This suggests that the U-HNP interface is formed by the polar heads of colistin and likely some phospholipidic choline segments. In contrast, the chitosan-coated hybrid nanoparticles (Ch-HNPs) obtained by the layer-by-layer coating method (Figure 3E) did not show any significant changes compared to the U-HNPs. The Z-average particle size was measured at approximately 251 nm, corresponding to a single population with a particle size of 211.6 nm ± 9.104 nm, a very low polydispersity index (PDI = ~0.150), and a zeta potential of +19.6 mV ± 1.5 mV. These results suggest the formation of a nanoparticulate system that retains the structural configuration of U-HNPs. This is particularly relevant given the characteristics of the single chitosan aqueous dispersion, which generates multiple polydisperse aggregates (PDI = ~0.842) with a high zeta potential value of +64.9 mV ± 3.230 mV. Therefore, the changes in PDI and zeta potential illustrate that the layer-by-layer coating was successfully achieved. The random coil-type chitosan aggregates disintegrated and migrated toward the surface of the U-HNPs, coating them in a controlled manner and generating a compact and highly organized layer. Furthermore, the significant change in zeta potential values—from +64.9 mV ± 3.230 mV (chitosan polymer) to +19.6 mV ± 1.5 mV (Ch-HNPs)—indicates that the surface coating is substantial. This change is likely due to the establishment of multiple polar interactions between the protonated D-glucosamine groups of chitosan and several polar groups of colistin and phosphatidylcholine.

#### 3.1.4. Characterization of HNPs by DSC and FTIR

**Figure 4:**
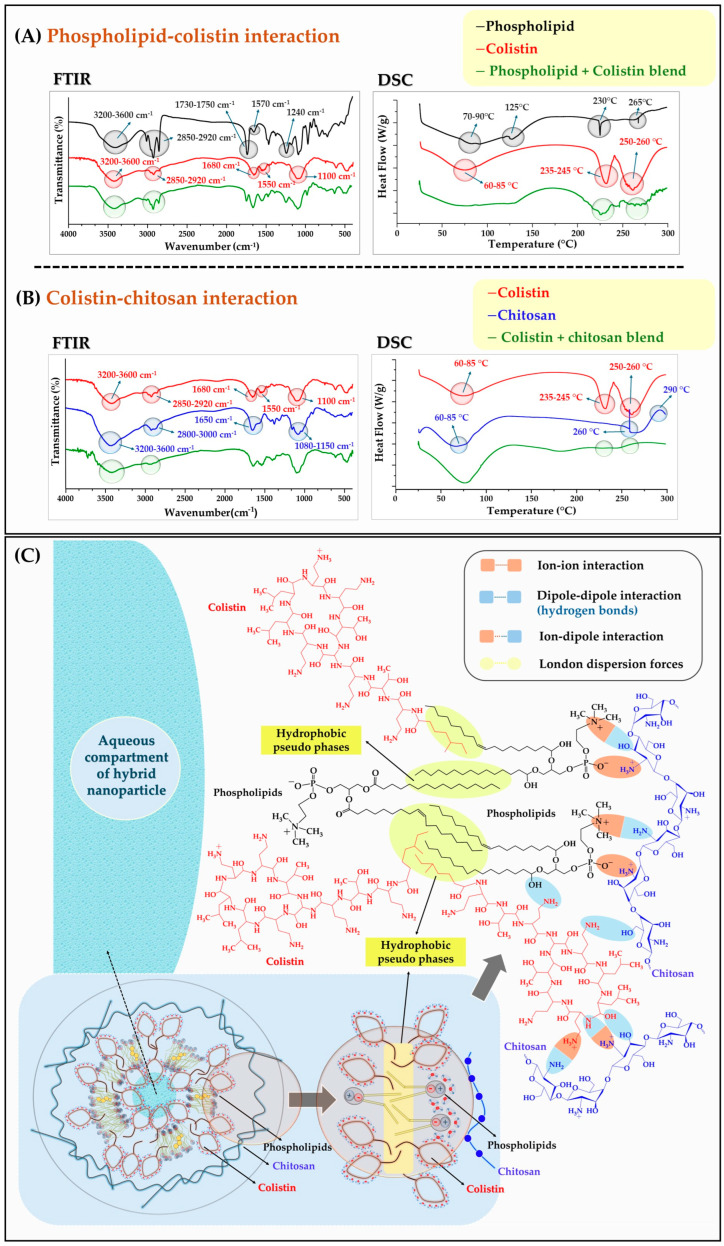
FT-IR and DSC characterization of the HNPs ingredients in a free form and a binary mixture. (**A**) Phospholipon^®^ 90G and Colistin. (**B**) Colistin and Chitosan. (**C**) Description of the intermolecular interactions given by the components of the HNPs.

The results of the DCS and FTIR characterization, along with a schematic representation of the potential interactions between the components of the HNPs, are displayed in Figure 4. In the case of pure Phospholipon^®^ (Figure 4A), the most prominent FTIR signals occurred in the following ranges: N-H and O-H stretching between 3200 cm^−^^1^ and 3600 cm^−^^1^, C-H stretching between 2850 cm^−^^1^ and 2920 cm^−^^1^, C=O stretching between 1730 cm^−^^1^ and 1750 cm^−^^1^, N-H bending at 1570 cm^−^^1^, and C-N stretching at 1240 cm^−^^1^. Additionally, the main DSC signals of phospholipid corresponded to a gel-to-liquid crystalline phase transition occurring between 70 °C and 90 °C. This transition can be attributed to the change from an organized to a disorganized lamellar structure [49]. An endothermic signal observed at 125 °C indicates the loss of water bound to phospholipid heads, while two distinct endothermic signals at 230 °C and 265 °C are attributed to the phase changes in Phospholipon^®^ compounds (Phosphatidylcholine and lysophosphatidylcholine) [50,51].

For pure colistin (Figure 4A), the most notable FTIR signals were observed in the following regions: an N-H and O-H stretching between 3200 cm^−^^1^ and 3600 cm^−^^1^, a C-H stretching between 2850 cm^−^^1^ and 2920 cm^−^^1^, a C=O stretching at 1680 cm^−^^1^, an N-H bending at 1550 cm^−^^1^, and a P-O-C stretching at 1100 cm^−^^1^. In contrast, the most significant DSC signals included a broad endothermic signal around 60–85 °C, which was attributed to the loss of water adsorbed on the surface of colistin. Additionally, a sharp endothermic signal was observed at 235–245 °C, corresponding to the melting of the peptide, and a further endothermic signal was detected at 250–260 °C, associated with the thermal decomposition of the molecule [52,53].

Regarding the pure chitosan polymer (Figure 4B), the most notable FTIR signals are associated with the following stretching vibrations: the N-H and O-H stretching observed in the range of 3200–3600 cm^−^^1^, the C-H stretching of the glucosamine ring between 2800 and 3000 cm^−^^1^, and the C=O stretching of the carbonyl group in the N-acetylglucosamine structure. Additionally, stretching of the C-O groups occurs in the range of 1080–1150 cm^−^^1^ [54]. In contrast, the primary DSC signals observed for chitosan include a broad endothermic signal ranging from 60 to 85 °C, which is attributed to the loss of surface water. Additionally, two exothermic signals are detected at 260 °C and 290 °C, associated with the advanced decomposition of the macromolecule [55].

In the case of binary blends, it is important to note that these were prepared using the same proportions employed during the development of the hybrid nanocarriers (HNPs). Additionally, the blends were dispersed in water and then dried through lyophilization and distillation at reduced pressure. The results from the mixture of phospholipid and colistin (Figure 4A) indicated that the FTIR signals for N-H and O-H stretching, which fall within the range of 3200 cm^−^^1^ to 3600 cm^−^^1^, were moderately attenuated. In contrast, the C-H stretching signals between 2850 cm^−^^1^ and 2920 cm^−^^1^ showed a considerable change in intensity. Differential Scanning Calorimetry (DSC) results demonstrated the disappearance of the signal associated with the loss of water adhered to the polar segment of the phospholipid. There was also a noticeable variation in the shape and intensity of signals at 230 °C and 265 °C, which correspond to the phase transition of the phospholipid. Similarly, signals at 235–245 °C and 250–260 °C related to the fusion and subsequent decomposition of colistin also exhibited changes. These alterations can be attributed to the formation of new dipole–dipole intermolecular interactions between the polar segments of Phospholipon^®^ and colistin, supporting the idea of a hybrid system formed by colistin and Phospholipon^®^ aggregates (Figure 4C).

Regarding the mixture of colistin and chitosan (Figure 4B), a change in the FTIR signal between 3200 cm^−^^1^ and 3600 cm^−^^1^ was also observed. This change may be due to the overlapping of −OH and −NH groups resulting from dipole–dipole interactions between the polar head of colistin and the chitosan polymer. Additionally, the disappearance of the signal associated with the loss of water adhered to the polar segment of Phospholipon^®^, coupled with a decrease in signal intensity at 235–245 °C and 250–260 °C related to the fusion and decomposition of colistin, suggests the formation of dipole–dipole interactions arising from the coating of chitosan on the surface of colistin (Figure 4C).

#### 3.1.5. Colistin Association and Release Efficiency and Morphology of HNPs

**Figure 5:**
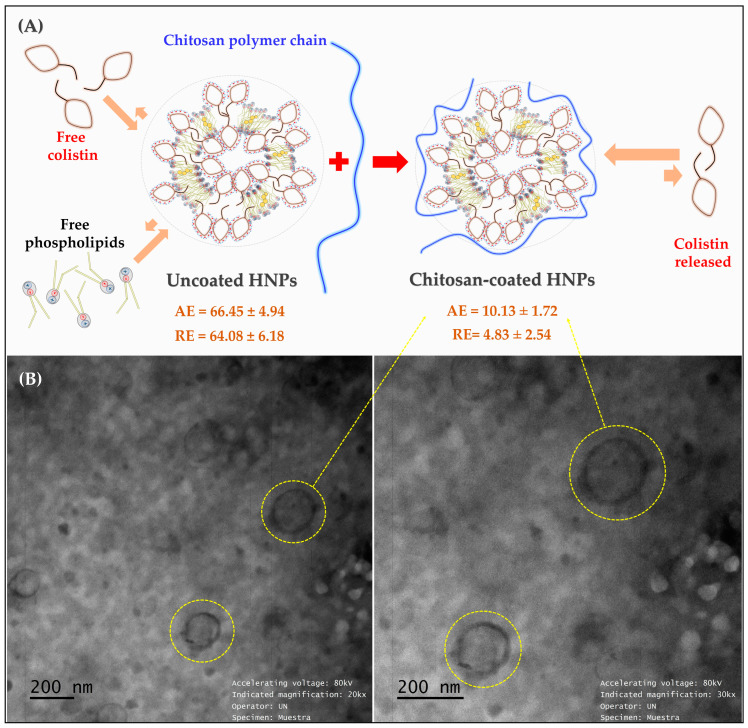
(**A**) Schematic of the mechanism of association and release of Colistin in HNPs. (**B**) TEM micrograph of HNPs consisting of phospholipids, cholesterol, Colistin, and Chitosan. AE: association efficiency of colistin. RE: Release efficiency of Colistin.

The association and release efficiency of colistin on U-HNPs and Ch-HNPs are detailed in Table 2 and illustrated in Figure 5A. Moreover, the morphology of the HNPs, observed through TEM microscopy, is presented in Figure 5B.

The results indicated that the association efficiencies for U-HNPs and Ch-HNPs were 66.45 ± 4.94 and 64.08 ± 6.18, respectively. These values were lower than the ~79% association efficiency that was initially achieved in the experimental design. This discrepancy can be attributed to the fact that the formation of hybrid nanoparticles involves several processes governed by multiple thermodynamic equilibria occurring simultaneously (Figure 5A). Consequently, the free forms of colistin and phospholipid can generate respective micellar and vesicular aggregates, which may compete with the formation of hybrid nanoparticles (HNPs). This finding suggests that the development of such nanosystems should be optimized and standardized in future studies.

It is important to note that the association efficiency values for U-HNPs and Ch-HNPs were very similar, demonstrating a degree of reproducibility in the development process. Additionally, these results clarify the observed low release efficiency (RE) of colistin, which was 10.13% ± 1.72% for U-HNPs and 4.83% ± 2.54% for Ch-HNPs. This suggests that colistin is more likely to remain in an aggregated form rather than in a free form. Moreover, it was noted that the RE for Ch-HNPs was approximately half that of that for U-HNPs, which can be explained by the presence of the chitosan polymer that extends along the surface of the nanoparticle, limiting the migration of colistin from the aggregated form to the free form. These findings are consistent with observations from surface tension and particle size studies, which also indicated a preference for aggregate forms.

On the other hand, electron microscopy revealed the presence of spherical systems about 200 nm in size, confirming the hypothesis of a highly organized nanoparticulate hybrid system, with characteristics like those reported for comparable aggregation systems, such as micelles and vesicles (Figure 5B). Thus, it can be concluded that under the conditions tested for HNP development, colistin is thermodynamically favored to form aggregates. These aggregates achieve greater stability when combined with phospholipid lamellar nanoaggregates and cholesterol, especially when coated with a chitosan film.

#### 3.1.6. Stability Assay of HNPs

**Figure 6:**
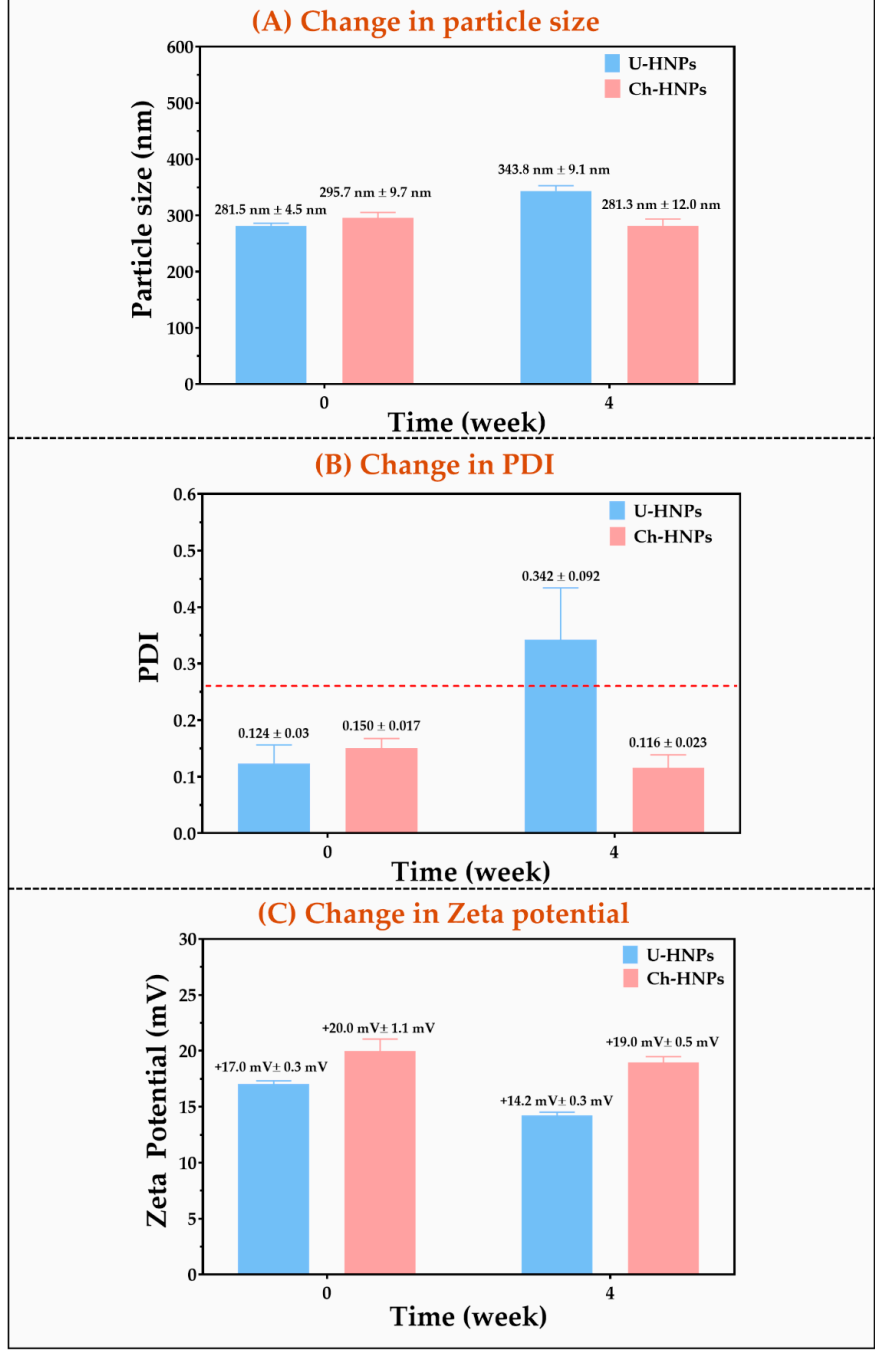
Results of the stability test of HNPs. (**A**) Particle size. (**B**) Polydispersity index. (**C**) Zeta potential.

The results of the stability assay for HNPs are presented in Figure 6. It was observed that the polymeric coating made with chitosan enhanced the physical stability of the nanoparticles, reducing their susceptibility to multi-aggregation processes that can occur in this type of nanostructured system [56,57]. The study found that the Z-average particle size for U-HNPs increased moderately from 281.5 nm ± 4.5 nm to 343.8 nm ± 9.1 nm. In contrast, the Z-average particle size for Ch-HNPs decreased slightly, from 295.7 nm ± 9.7 nm to 281.3 nm ± 12.0 nm (see Figure 6A). Regarding the polydispersity index (PDI), U-HNPs exhibited a moderate increase from 0.124 ± 0.033 to 0.342 ± 0.092. Conversely, the PDI for Ch-HNPs decreased slightly, from 0.150 ± 0.017 to 0.116 ± 0.023 (refer to Figure 6B). When examining the zeta potential, the value for U-HNPs decreased slightly from +17.0 mV ± 0.3 mV to +14.2 mV ± 0.3 mV. In contrast, the zeta potential for Ch-HNPs remained relatively constant, ranging between +20.0 mV ± 1.1 mV and +19.0 mV ± 0.5 mV (see Figure 6C). These results indicate that the stability of U-HNPs is moderate, evidenced by an increase in Z-average particle size of approximately 62 nm. This suggests a possible migration of the various components of the HNPs (colistin and phospholipid) toward other pre-formed aggregates or through inter-particle aggregation, which may occur via flocculation or coagulation processes [58]. This last statement makes more sense when the change in PDI is analyzed, which goes from a value representative of monodisperse systems (PDI < 0.3) to a value suggesting polydisperse systems (PDI > 0.3). Likewise, the zeta potential also described a slight decrease that may suggest a migration of colistin from the interfacial zone as previously mentioned. On the contrary, Ch-HNPs showed an appropriate stabilization behavior, where the coating polymer led to a slight compaction of the HNPs and was most likely given by the limitation of the migration of its components leading to a population of homogeneous size with practically constant interfacial characteristics. These results are consistent with the chemical nature of the compounds that make up these hybrid nanostructured systems. For example, colistin and phosphatidylcholine (the main component of Phospholipon^®^ 90G) have an amphiphilic character, as indicated in Section 3.1.1. Thus, the non-polar segments of both compounds in aqueous media are influenced by the hydrophobic effect, which is a very unfavorable thermodynamic condition due to the loss of entropy of water molecules (dispersing medium), which are adjacent to the hydrocarbon segments of these amphiphilic molecules.

In this manner, when these amphiphilic molecules are dispersed in aqueous media, a process of multiple thermodynamic equilibria occurs, where the free molecules begin to self-assemble, forming different types of structures. In the case of colistin, which has a large polar head and a very small tail (Figure 2A), it tends to form micellar aggregates, with a small non-polar core and a large, slightly compacted polar head. In contrast, for phosphatidylcholine, which has two alkyl chains (Figure 2B), the self-assembly process takes place through the aggregation of a bilayer or lamellae, which in turn aggregates with each other, forming vesicular structures. This tendency towards the formation of continuous equilibria of free forms to different aggregated forms can lead to the formation of different self-assembled systems, where even the aggregation of other types of systems made up of both colistin and Phospholipon^®^ 90G can occur. However, the coating with chitosan polymer significantly influences such aggregation conditions, affecting the molecular migration and directing self-assembly towards nanostructured systems that maintain particle size, polydispersity, and zeta potential for longer.

### 3.2. Biological Evaluation

#### 3.2.1. Isolation of Resistant *K. pneumoniae* and Phenotypic Characterization

Table 3 presents the phenotypes of two *K. pneumoniae* strains used in this study. These strains are categorized as extensively drug-resistant, as they are only susceptible to the aminoglycoside class of antibiotics [59]. Both strains produce enzymes that can degrade the beta-lactam ring, allowing them to evade the action of inhibitors like TZP, CAZ, and FEP. They are also capable of producing carbapenemases, which inactivate carbapenem antibiotics such as DOR, IPM, and MEM. Additionally, these strains can reduce their affinity for quinolones (CIP) through modifications to DNA gyrase, an essential enzyme for DNA replication, thereby evading the effects of this antibiotic [60]. Finally, resistance to lipopeptides (CST) indicates that these strains can neutralize the surface anionic charge through structural modifications, a topic that will be discussed further.

#### 3.2.2. Antimicrobial Activity

Initially, the antimicrobial effect of chitosan on clinical isolates Kp-01 and Kp-02 was evaluated, where it was observed that it does not have a considerable effect exhibiting a MIC of 1500 µg/mL. However, it would be negligent to ignore a slight inhibitory effect. Considering this result, Tamer et al. [61] has suggested three scenarios to address the possible antibacterial mechanism of action of chitosan: First, the protonated NH_3_^+^ groups interact with the anionic components of the cell wall promoting the release of cytoplasmic content; second, the cationic chitosan would have the ability to cross the bacterial surface and interact with the phosphate groups of DNA altering the cellular transcription and translation processes; third, the chelating properties of chitosan facilitate the retention of metal ions, which are essential elements for the growth of bacterial cells.

**Figure 7:**
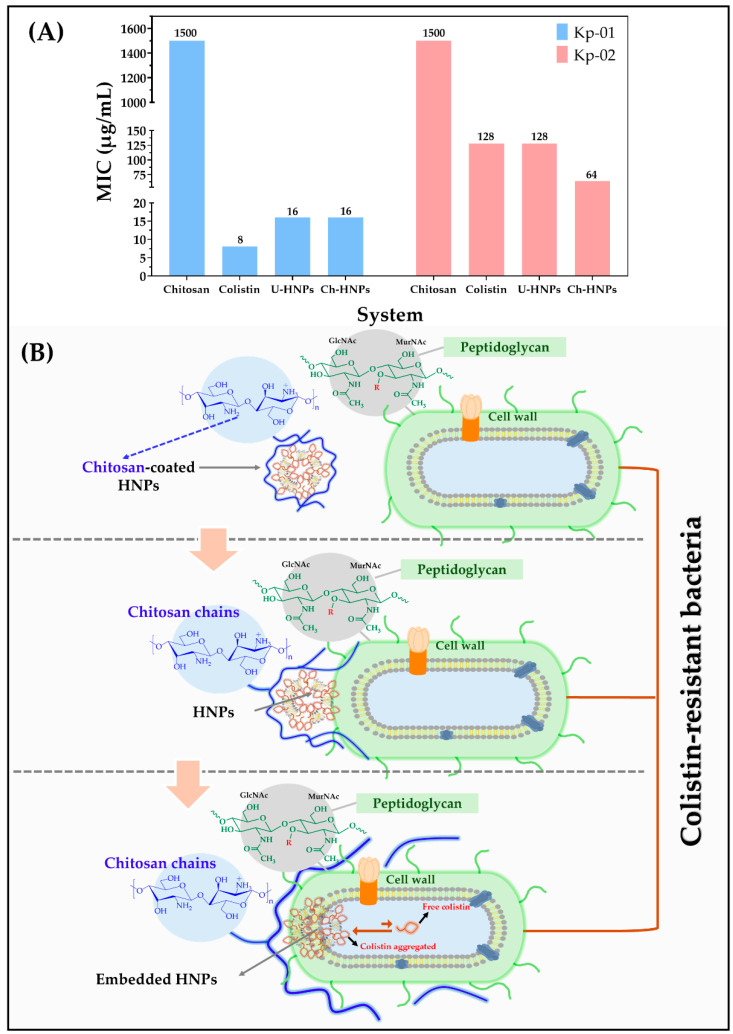
Antimicrobial activity of pure colistin and as part of uncoated (U-HNPs) and chitosan-coated (Ch-HNPs) hybrid nanoparticles. (**A**) Minimum inhibitory concentration (MIC) results. (**B**) Representative scheme of the mechanism of action of Ch-HNPs on the surface of *K. pneumoniae*.

On the other hand, the results of the antimicrobial activity of hybrid nanoparticles (HNPs) containing colistin, Phospholipon^®^, cholesterol, and chitosan are illustrated in Figure 7. The findings indicate that the antibacterial activity of pure colistin against resistant *K. pneumoniae* strains demonstrated a minimum inhibitory concentration (MIC) of 8 μg/mL for strain Kp-01 and 128 μg/mL for strain Kp-02. These MIC values are above the sensitivity breakpoint (MIC ≤ 2 μg/mL) as defined by EUCAST [62]. To understand these results, it is important to note that resistance to colistin primarily arises from the neutralization of the anionic charge of lipid A in the lipopolysaccharide found on the bacterial surface. This charge interacts electrostatically with colistin, which possesses a slightly positive surface character, as confirmed by previous studies (Figure 7B) [63]. The modification occurs due to the addition of 4-amino-4-deoxy-L-arabinose to the phosphate group of lipid A, a process mediated by the gene products of the *arnBCADTEF* operon. This operon is positively regulated by the two-component system PhoP/PhoQ, which is inhibited by the MgrB protein [64]. In colistin-resistant *K. pneumoniae*, mutations in the mgrB gene have been directly linked to resistance, as previously suggested [65]. Furthermore, insertional inactivation mediated by mobile DNA is described in the literature as the most prevalent mechanism contributing to colistin resistance in *K. pneumoniae* [66]. The most reported mgrB mutations associated with this resistance include the mgrBCys28Tyr substitution, as well as mgrBCys28* and mgrBGln30* deletions [67]. Interestingly, strain Kp-02 requires a substantially higher concentration of colistin to inhibit its growth compared to Kp-01. Although the genomes of both strains are not accessible for mutation analysis, the difference in MICs may be attributed to the fact that resistance to colistin can result from two or more mechanisms [66]. This indicates that strain Kp-02 may possess more deleterious mutations in the mgrB gene that lead to the upregulation of PhoP-regulated genes. Additionally, different mutations in this strain could collectively enhance its resistance against the action of colistin.

On another note, the results for colistin as a component of HNPs did not show significant changes in antimicrobial activity. Specifically, for strain Kp-01, the MIC increased from 8 μg/mL to 16 μg/mL for both U-HNPs and Ch-HNPs. In contrast, for strain Kp-02, there was no change in the MIC for U-HNPs, remaining at 128 μg/mL, while for Ch-HNPs, the MIC decreased to 64 μg/mL. These observations can be explained by considering that the major component of HNPs is colistin, which tends to form micellar aggregates, as discussed in Section 3.1.1.

Moreover, these antimicrobial outcomes may also relate to the association and release efficiencies found in this study, where there was a notable tendency toward association (AE > 60%) and a poor tendency toward release (RE < 10%). Colistin’s self-assembly characteristics limit its free-form availability, significantly impacting its antimicrobial effectiveness. This observation suggests that, as noted in previous studies [67], colistin is either less accessible or is released in a controlled manner. Additionally, it is essential to highlight that the coating of hybrid nanoparticles (HNPs) with chitosan polymer did not significantly affect antimicrobial activity, suggesting that the slight antimicrobial effect of free chitosan reported above was not sufficient to contribute to the biological activity of HNPs. In addition, these results are contrary to expectations based on literature regarding the bioadhesive properties of this material (Figure 7A) [68]. However, these findings can be understood considering research by Kumar et al. [69], who indicated that low molecular weight chitosan polymers exhibit more significant antimicrobial activity than those with a higher molecular weight, like those used in this study. Moreover, Chung et al. [68] showed that reducing the polymer size enhances selectivity toward Gram-negative bacteria while having the opposite effect on Gram-positive bacteria. In support of this, Campana et al. [70] found that 50 kDa chitosan demonstrated more significant antibacterial activity against clinical isolates of *K. pneumoniae* than 150 kDa chitosan. These findings pave the way for future research on the time-dependent evaluation of these hybrid nanoparticulate systems using chitosan of varying molecular weights. Furthermore, exploring the effects of HNPs loaded with conventional antibiotics on highly antibiotic-resistant bacteria would be intriguing.

## 4. Conclusions

Colistin and Phospholipon^®^ in an aqueous medium displayed high surface activity, indicating a tendency to form thermodynamically favorable molecular aggregates with monodisperse characteristics in the nanometric range. Colistin tended to form micelle-type aggregates, while Phospholipon primarily formed small unilamellar vesicles (SUVs). In contrast, aqueous chitosan dispersions show poor surface activity, as chitosan tends to be solubilized in the aqueous medium, forming random coil-type aggregates depending on its concentration.

The HNPs were developed to comprise a combination of peptide micellar fragments from colistin intertwined with vesicular lipid bilayer fragments from Phospholipon^®^. These nanoparticles created monodisperse populations with slightly polarized interfaces that can be easily coated with a high molecular weight chitosan polymer. This coating establishes multiple ion–dipole and dipole–dipole surface interactions, forming highly ordered and compact nanoaggregates.

Additionally, these HNPs maintain their physical and morphological characteristics over time, preventing disintegration and the migration of their components into the aqueous dispersion medium. This stability limits colistin’s performance since poorly released colistin restricts the optimal concentration needed to achieve its pharmacodynamic effect. Therefore, further studies are required to explore how the antimicrobial effectiveness of these nanoparticles changes over time and how these may enhance the effects of conventional antibiotics when used in conjunction with these hybrid nanoparticles against resistant strains of microorganisms.

## Figures and Tables

**Table 1:** Results of 2^k^ (k = 3) factorial design for the optimization of HNPs.

Run	Colistin (mg/mL)	Phospholipon^®^ 90G (mg/mL)	Cholesterol (mg/mL)	Particle Size (nm)	PDI	AE (%)
1	5	3	0.45	244.3	0.045	33.9
2	10	3	0.90	208.6	0.025	70.8
3	5	3	0.90	232.2	0.063	54.0
4	10	1.5	0.23	220.2	0.262	78.7
5	10	3	0.45	236.3	0.052	68.4
6	5	1.5	0.23	484.7	0.678	22.7
7	5	1.5	0.45	306.5	0.188	34.1

**Table 2:** Results of the association and release efficiency of colistin in HNPs.

System	Association Efficiency (AE) (%)	Release Efficiency (RE) (%)
U-HNPs	66.45 ± 4.94	10.13 ± 1.72
Ch-HNPs	64.08 ± 6.18	4.83 ± 2.54

U-HNPs: Uncoated hybrid nanoparticles; Ch-HNPs: Chitosan-coated hybris nanoparticles.

**Table 3:** Bacterial resistance profile from phenotypic tests.

Strain	MIC (ug/mL)									
	TZP	CAZ	FEP	DOR	IPM	MEM	AMK	GEN	CIP	CST
Kp-01	≥128/R	≥64/R	≥64/R	≥8/R	≥16/R	≥16/R	≥16/S	≥8/I	≥4/R	≥8/R
Kp-02	≥128/R	≥64/R	≥64/R	≥8/R	≥16/R	≥16/R	≥8/S	≥1/S	≥0.2/R	≥64/R

*Kp*: *K. pneumoniae*; S: Susceptibility strain; R: Resistant strain.

## Data Availability

Data are available in the manuscript.

## Supplementary Materials

The following supporting information can be downloaded at: https://www.mdpi.com/article/10.3390/pharmaceutics17020182/s1, where a supplementary file is provided with all recorded data on particle size and zeta potential obtained through light scattering and capillary electrophoresis, ensuring the quality of each measurement.
