# Supplementary material for: Development, Characterization, and Antimicrobial Evaluation of Hybrid Nanoparticles (HNPs) Based on Phospholipids, Cholesterol, Colistin, and Chitosan Against Multidrug-Resistant Gram-Negative Bacteria
Source: Pharmaceutics. 2025 Feb 1;17(2):182. doi: 10.3390/pharmaceutics17020182 (PMC11859251; doi:10.3390/pharmaceutics17020182)

Article

Development, Characterization, and Antimicrobial Evaluation of Hybrid Nanoparticles (HNPs) Based on Phospholipids, Cholesterol, Colistin, and Chitosan Against Multidrug-Resistant Gram-Negative Bacteria

Supplementary material

Particle size and PDI data

(A) Colistin aqueous dispersion (0.29 mM)

Temperature (°C):	25,0	Duration Used (s):	60
Count Rate (kcps):	293,4	Measurement Position (mm):	4,65
Cell Description:	Glass cuvette with round aperture	Attenuator:	10

Correlogram Report

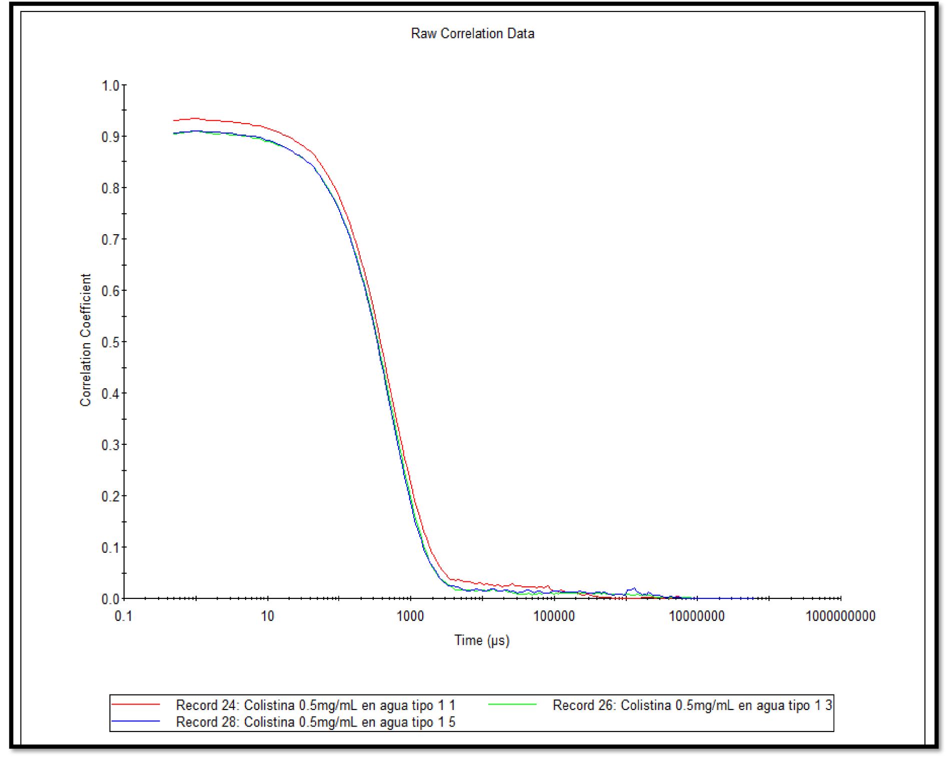

Size Distribution Report by Number

				Size (d.nm):	% Number:	St Dev (d.nm):
Z-Average (d.nm):	367,3		Peak 1:	241,4	100,0	113,7
PdI:	0,232		Peak 2:	0,000	0,0	0,000
Intercept:	0,948		Peak 3:	0,000	0,0	0,000
Result quality :	Good					

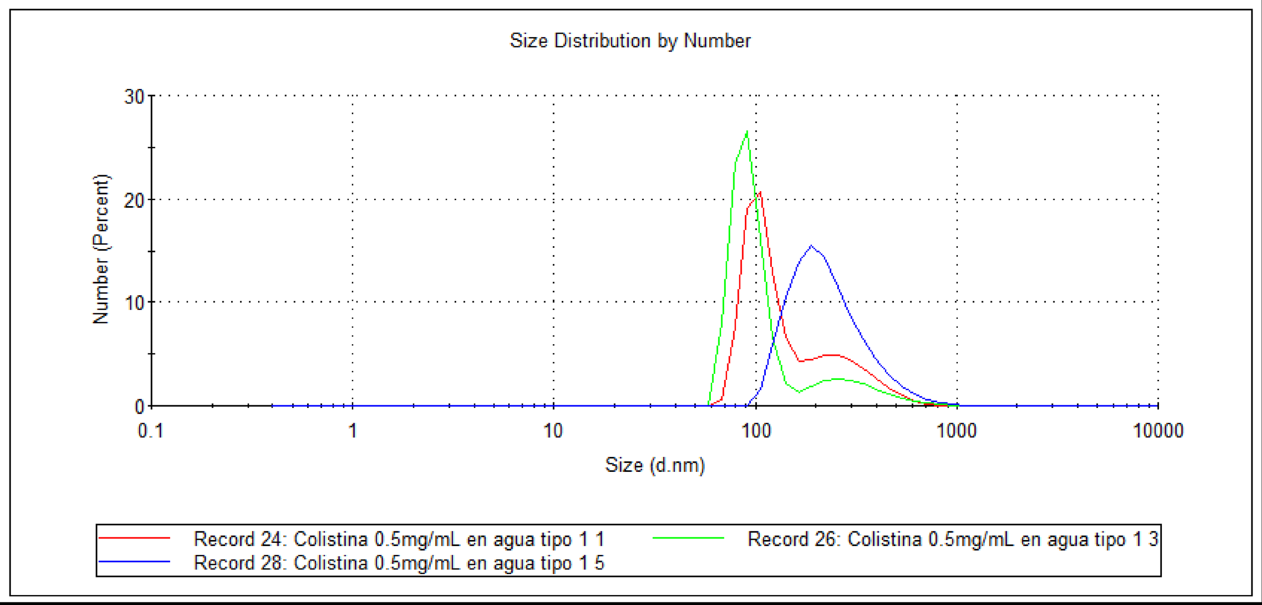

Size Distribution Report by intensity

				Size (d.nm):	% Intensity:	St Dev (d.nm):
Z-Average (d.nm):	367,3		Peak 1:	411,1	98,1	181,7
PdI:	0,232		Peak 2:	5203	1,9	473,2
Intercept:	0,948		Peak 3:	0,000	0,0	0,000
Result quality :	Good					

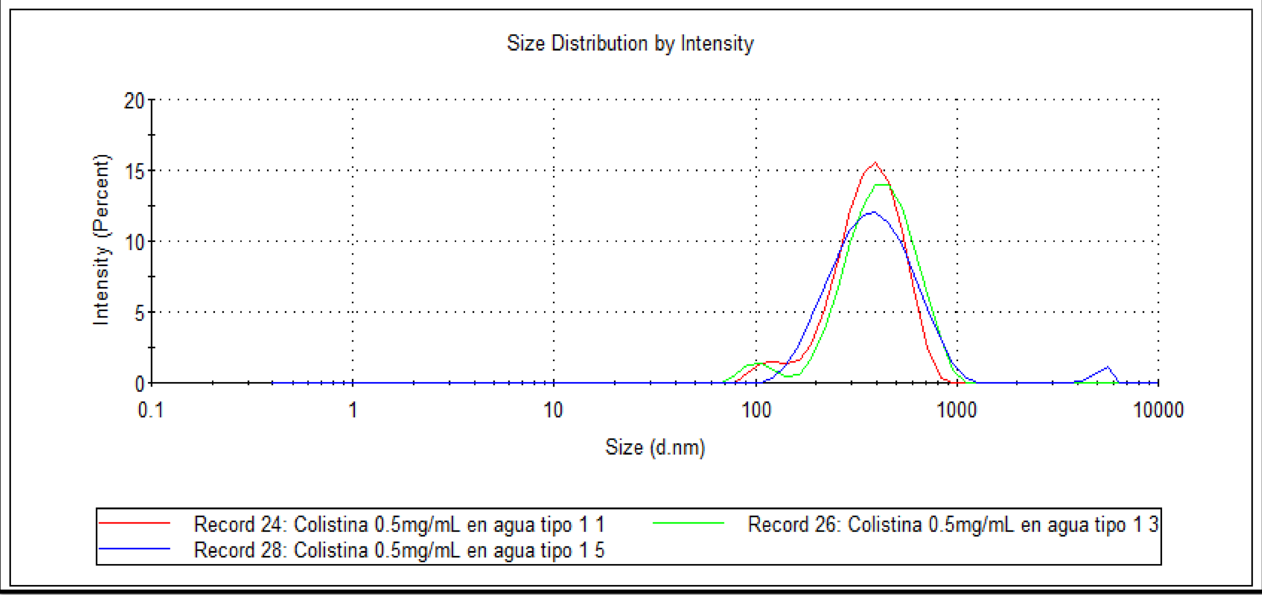

Size Distribution Report by Volume

				Size (d.nm):	% Volume:	St Dev (d.n...
Z-Average (d.nm):	367,3		Peak 1:	446,7	94,0	203,0
PdI:	0,232		Peak 2:	5347	6,0	690,2
Intercept:	0,948		Peak 3:	0,000	0,0	0,000
Result quality :	Good					

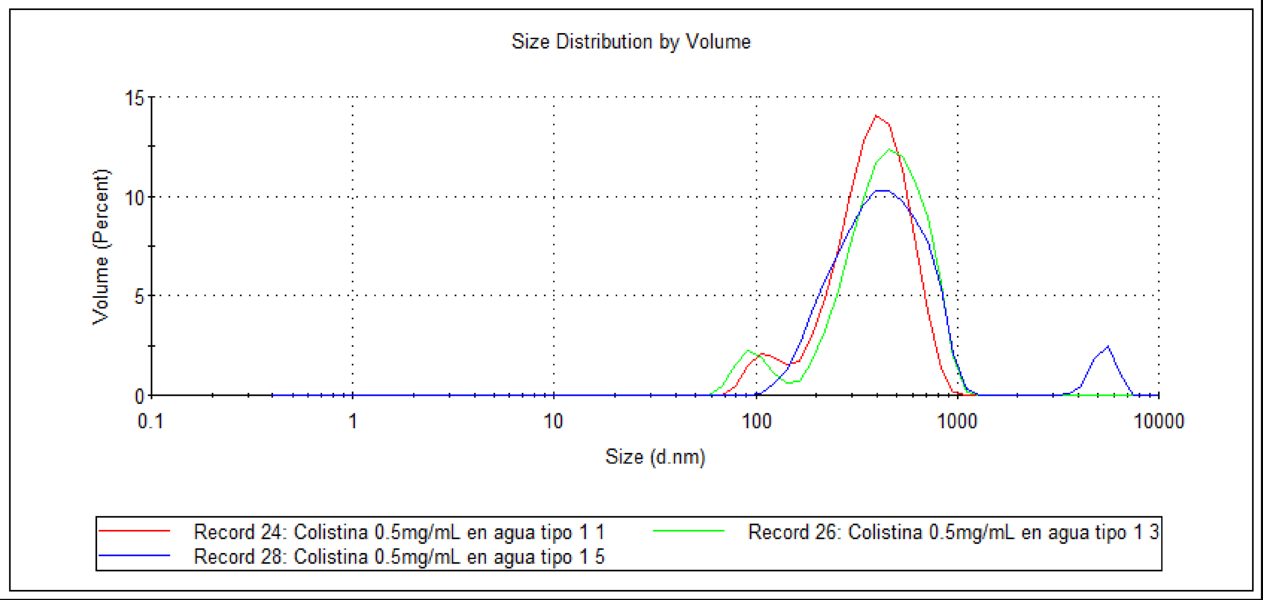

(B) Phospholipon ® aqueous dispersion (0.06 mM)

Temperature (°C):	25,0	Duration Used (s):	70
Count Rate (kcps):	175,3	Measurement Position (mm):	4,65
Cell Description:	Disposable sizing cuvette	Attenuator:	9

Correlogram Report

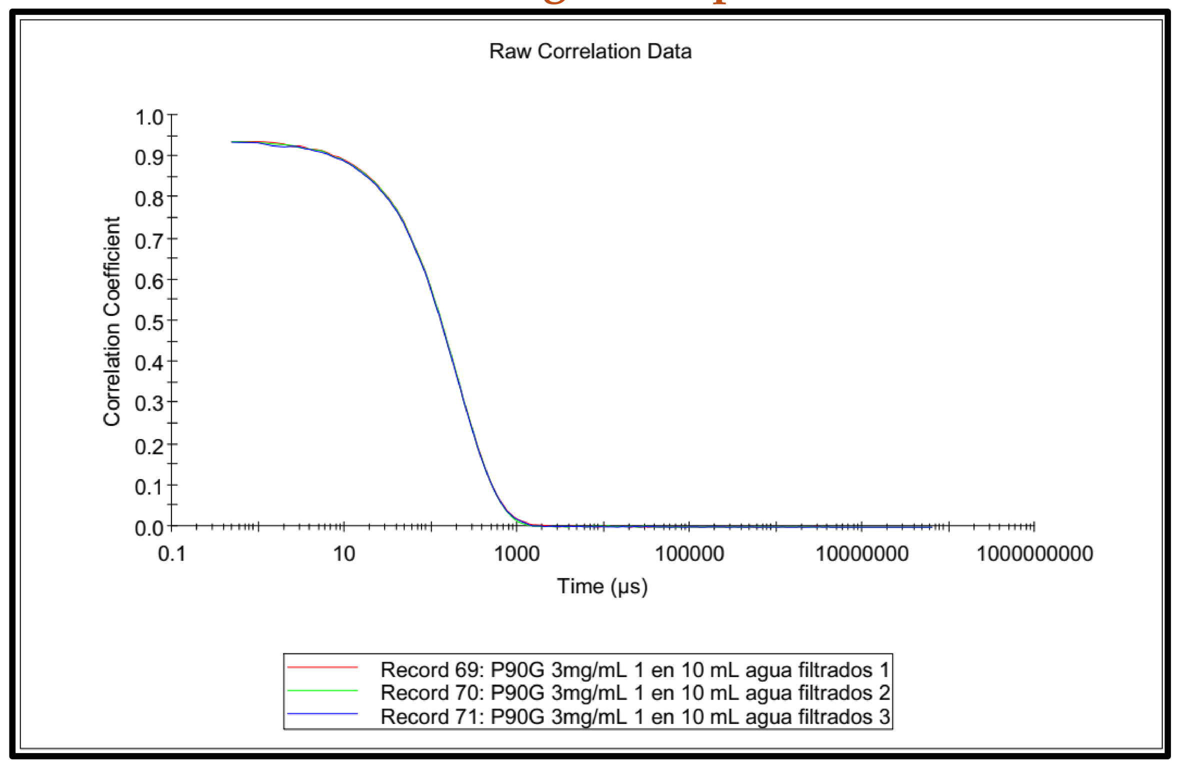

Size Distribution Report by Number

				Size (d.nm):	% Number:	St Dev (d.n...
Z-Average (d.nm):	100,6		Peak 1:	54,30	100,0	18,42
PdI:	0,182		Peak 2:	0,000	0,0	0,000
Intercept:	0,967		Peak 3:	0,000	0,0	0,000
Result quality :	Good					

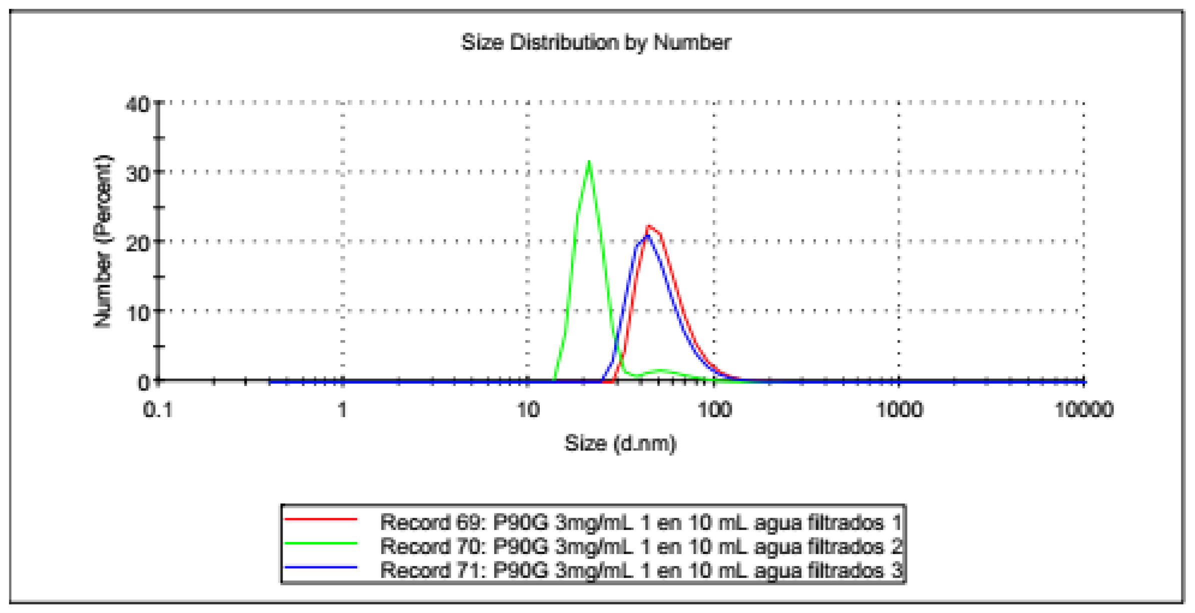

Size Distribution Report by intensity

				Size (d.nm):	% Intensity:	St Dev (d.n...
Z-Average (d.nm):	100,6		Peak 1:	120,9	100,0	54,85
PdI:	0,182		Peak 2:	0,000	0,0	0,000
Intercept:	0,967		Peak 3:	0,000	0,0	0,000
Result quality :	Good					

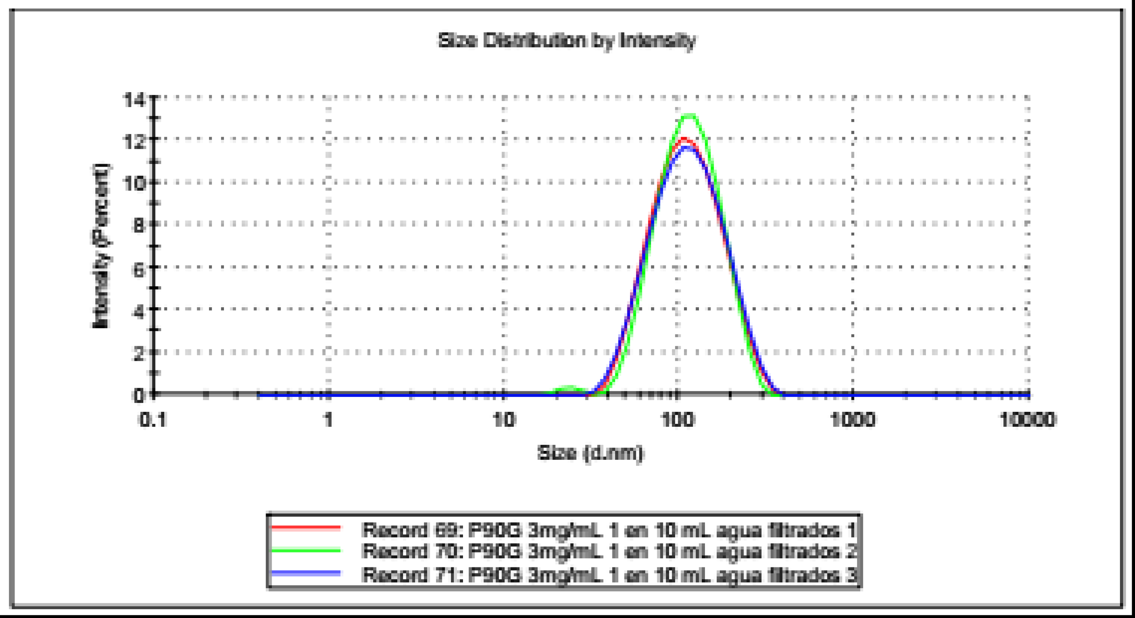

Size Distribution Report by Volume

				Size (d.nm):	% Volume:	St Dev (d.nm):
Z-Average (d.nm):	100,6		Peak 1:	84,33	100,0	47,21
PdI:	0,182		Peak 2:	0,000	0,0	0,000
Intercept:	0,967		Peak 3:	0,000	0,0	0,000
Result quality :	Good					

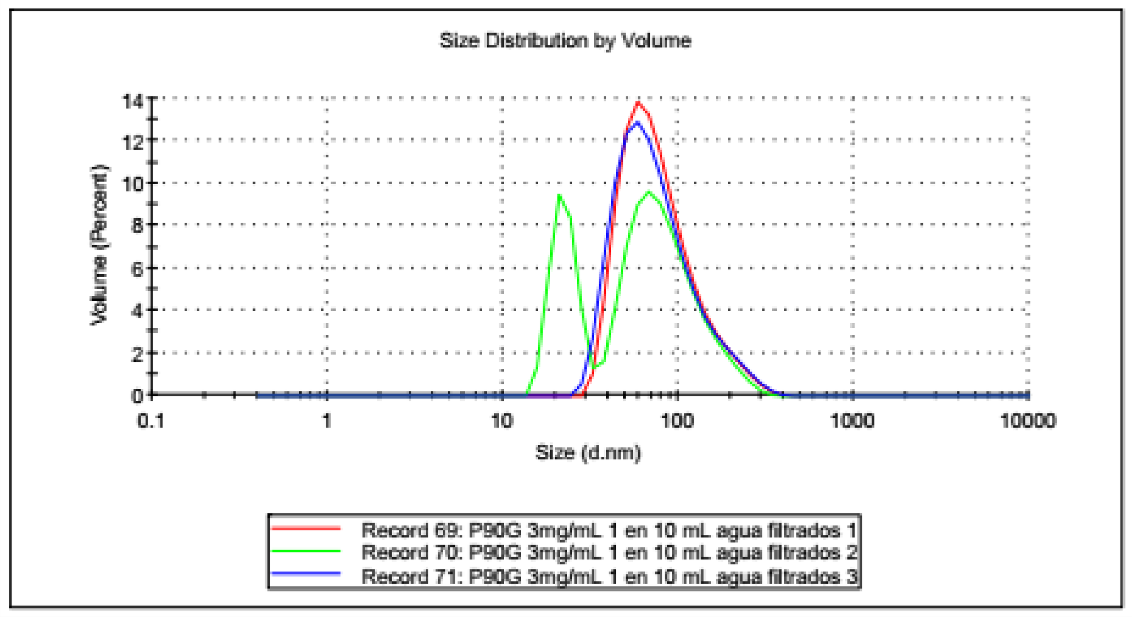

(C) Chitosan aqueous dispersion (1.0 mM)

Temperature (°C):	25,0	Duration Used (s):	14
Count Rate (kcps):	51,3	Measurement Position (mm):	4,65
Cell Description:	Disposable sizing cuvette	Attenuator:	11

Correlogram Report

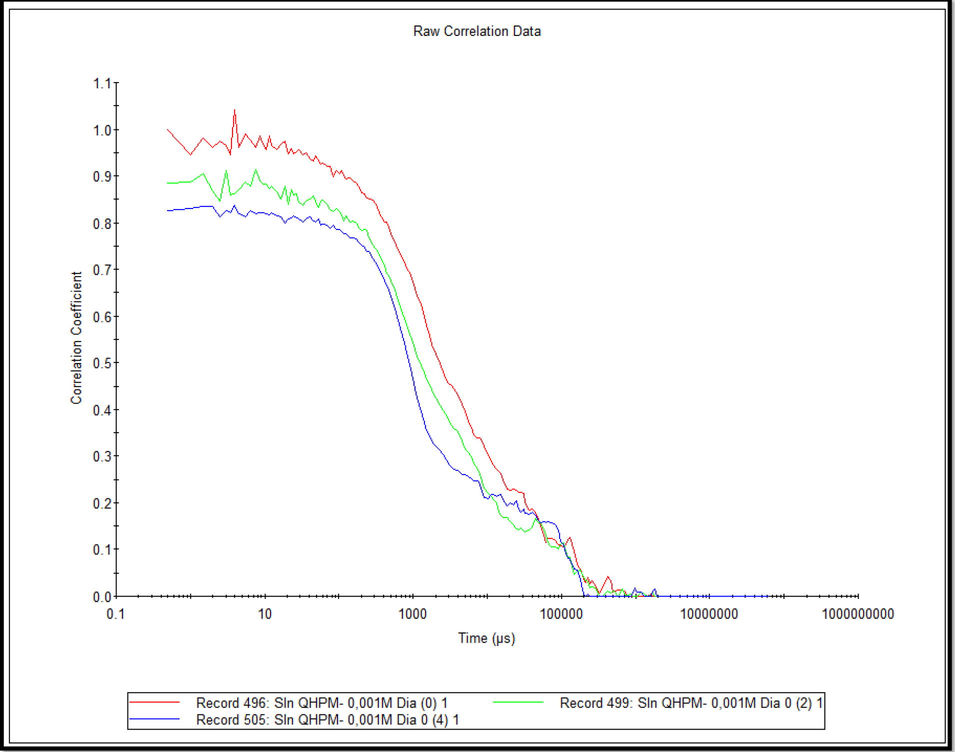

Size Distribution Report by Number

				Size (d.nm):	% Number:	St Dev (d.nm):
Z-Average (d.nm):	1010		Peak 1:	422,4	100,0	53,54
PdI:	0,721		Peak 2:	0,000	0,0	0,000
Intercept:	0,874		Peak 3:	0,000	0,0	0,000
Result quality :	Refer to quality report					

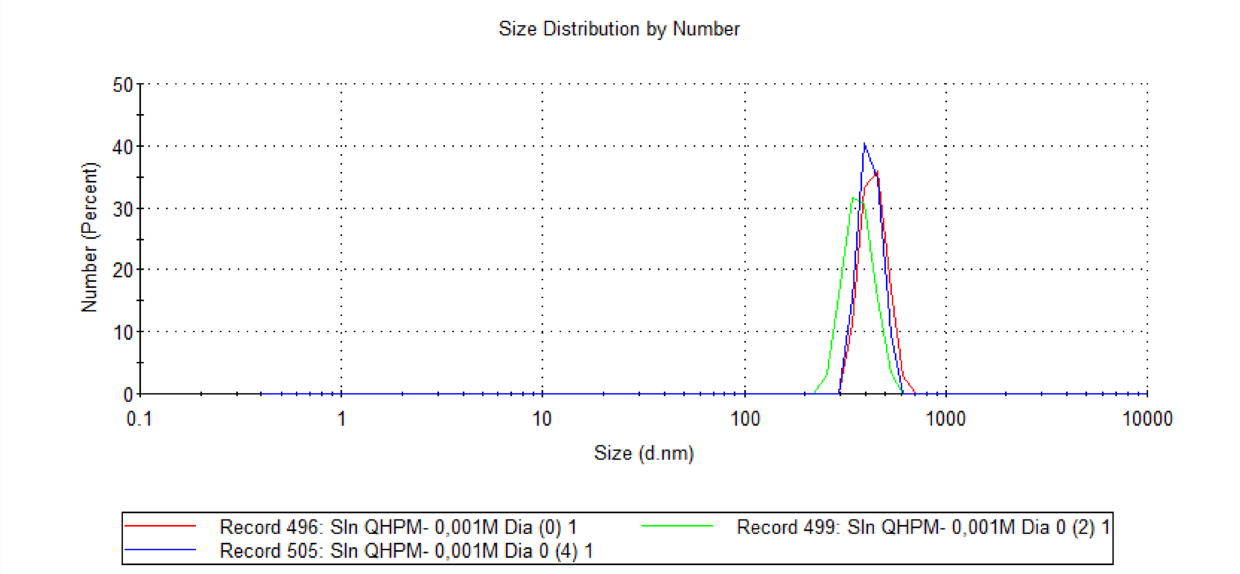

Size Distribution Report by intensity

				Size (d.nm):	% Intensity:	St Dev (d.nm):
Z-Average (d.nm):	1010		Peak 1:	425,1	100,0	31,23
PdI:	0,721		Peak 2:	0,000	0,0	0,000
Intercept:	0,874		Peak 3:	0,000	0,0	0,000
Result quality :	Refer to quality report					

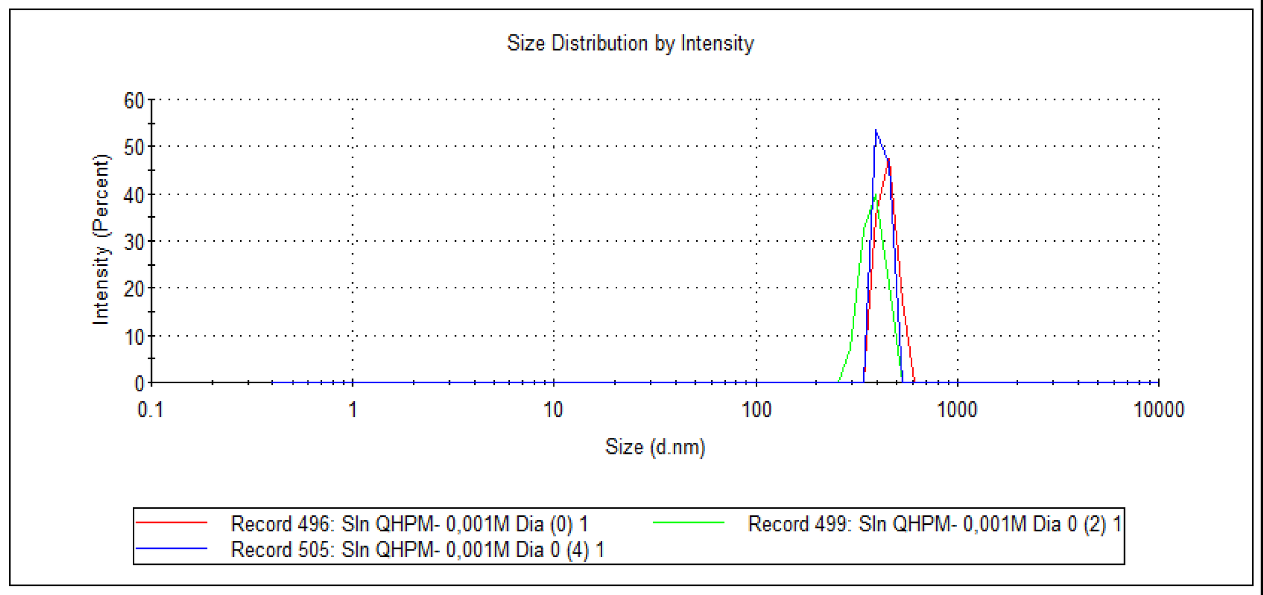

Size Distribution Report by Volume

				Size (d.nm):	% Volume:	St Dev (d.n...
Z-Average (d.nm):	1010		Peak 1:	429,2	100,0	54,61
PdI:	0,721		Peak 2:	0,000	0,0	0,000
Intercept:	0,874		Peak 3:	0,000	0,0	0,000
Result quality :	Refer to quality report					

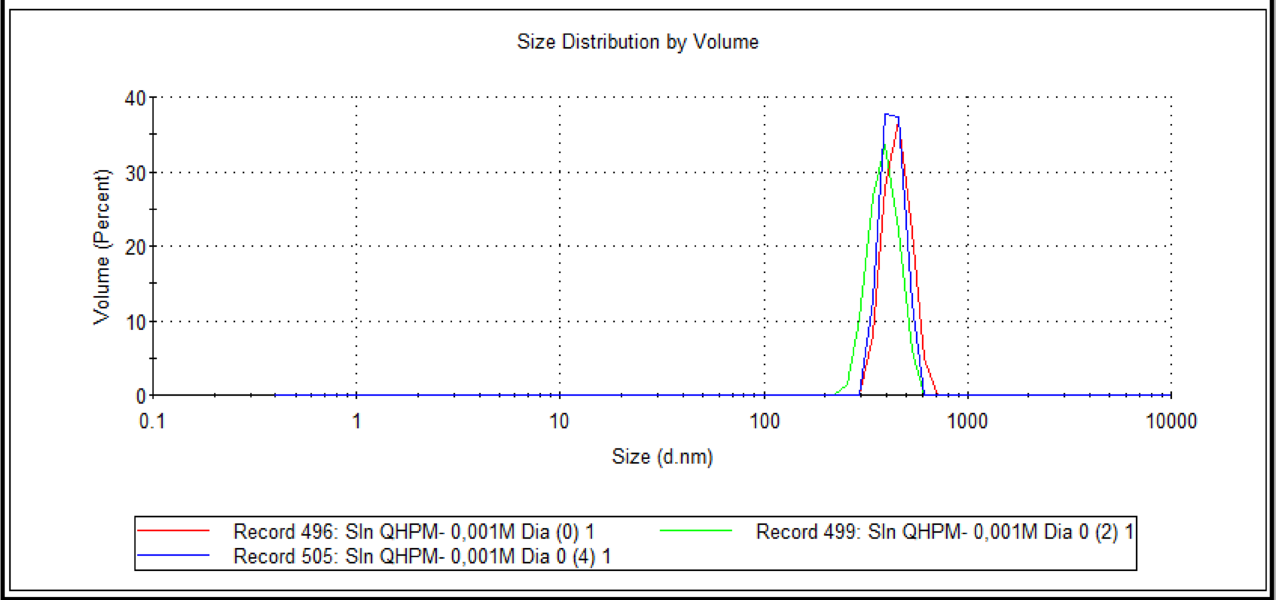

(D) U-HNPs

Temperature (°C):	25,0	Duration Used (s):	45
Count Rate (kcps):	486,8	Measurement Position (mm):	4,65
Cell Description:	Disposable sizing cuvette	Attenuator:	8

Correlogram Report

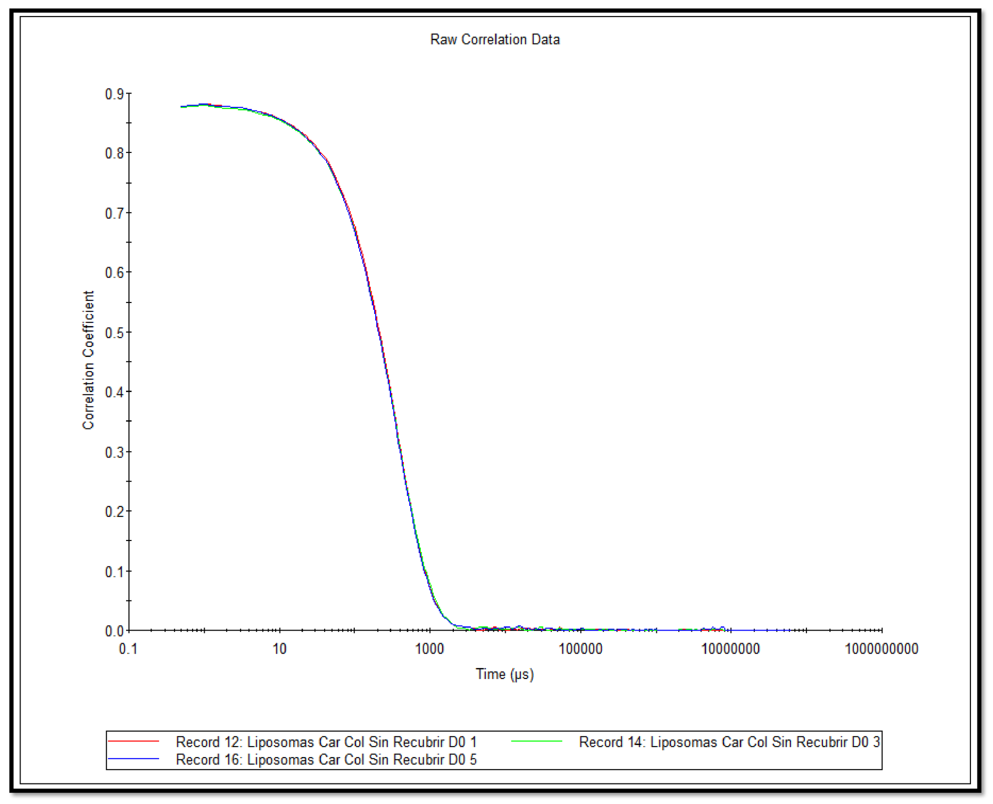

Size Distribution Report by Number

			Size (d.nm):	% Number:	St Dev (d.nm):
Z-Average (d.nm):	245,5	Peak 1:	205,1	100,0	80,88
PdI:	0,120	Peak 2:	0,000	0,0	0,000
Intercept:	0,939	Peak 3:	0,000	0,0	0,000
Result quality :	Good				

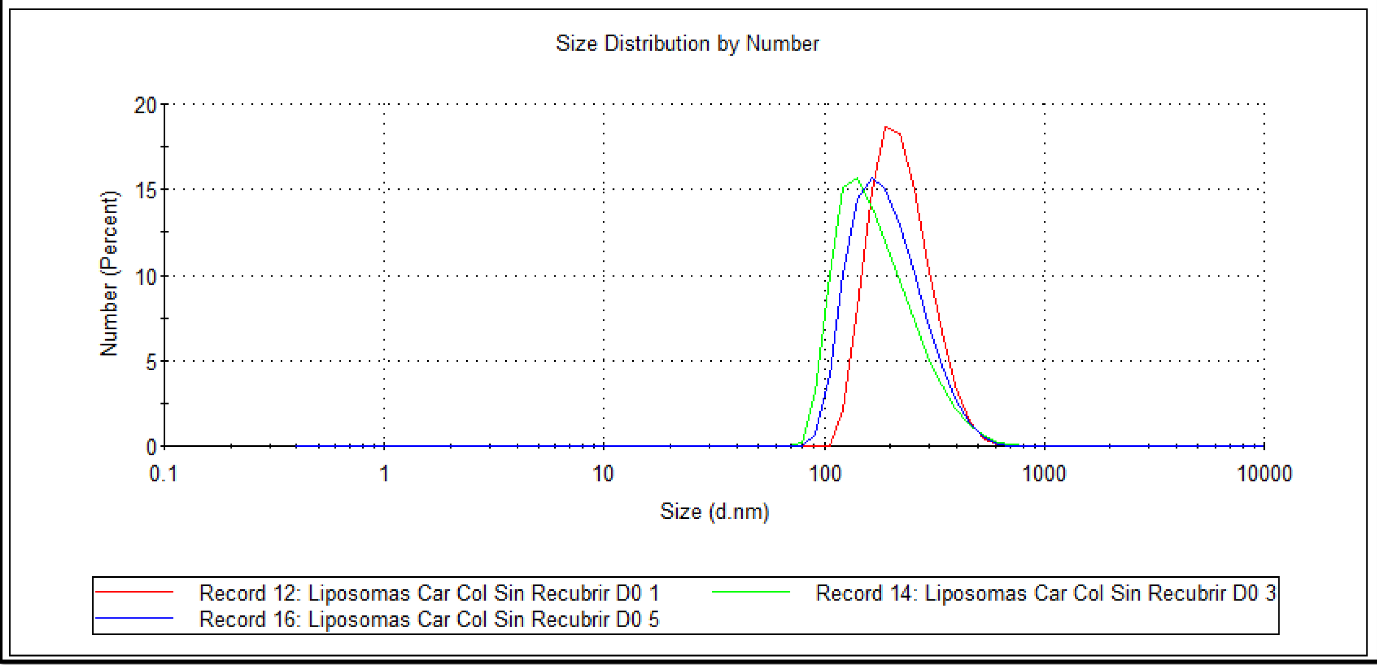

Size Distribution Report by intensity

			Size (d.nm):	% Intensity:	St Dev (d.nm):
Z-Average (d.nm):	245,5	Peak 1:	279,7	100,0	100,8
PdI:	0,120	Peak 2:	0,000	0,0	0,000
Intercept:	0,939	Peak 3:	0,000	0,0	0,000
Result quality :	Good				

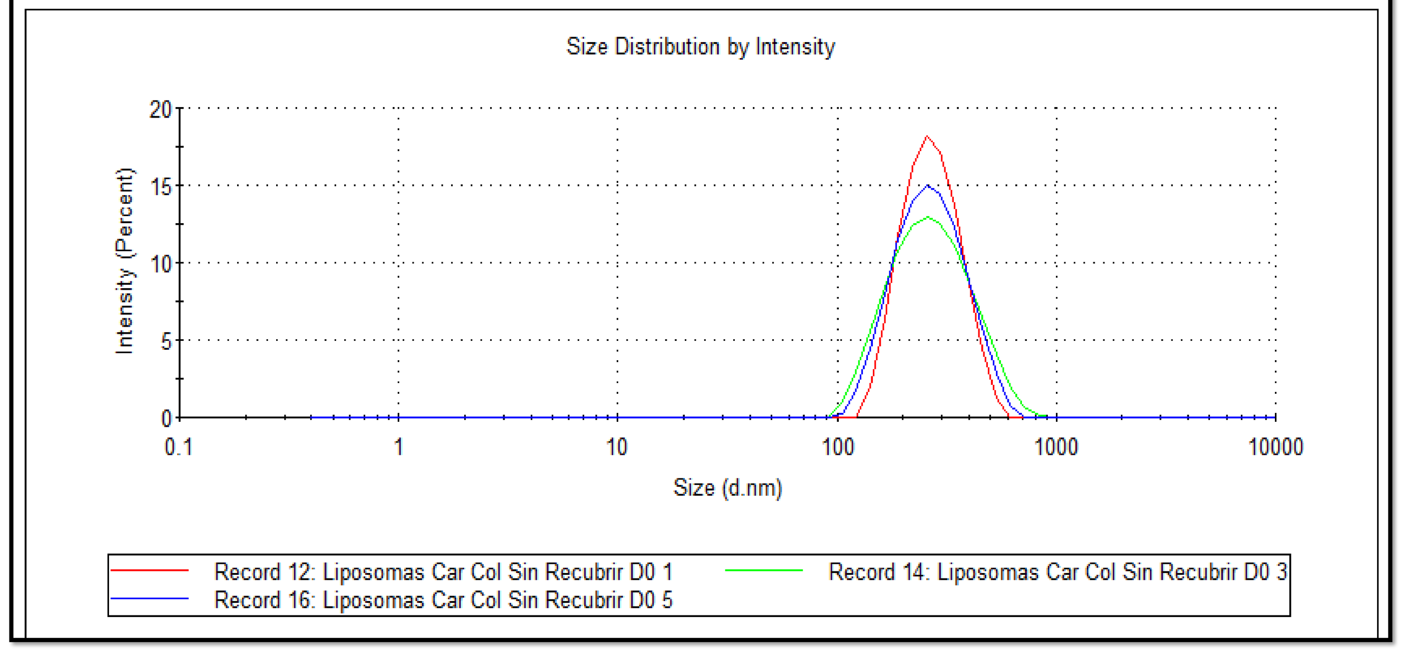

Size Distribution Report by Volume

			Size (d.nm):	% Volume:	St Dev (d.n...
Z-Average (d.nm):	245,5	Peak 1:	308,9	100,0	115,5
PdI:	0,120	Peak 2:	0,000	0,0	0,000
Intercept:	0,939	Peak 3:	0,000	0,0	0,000
Result quality :	Good				

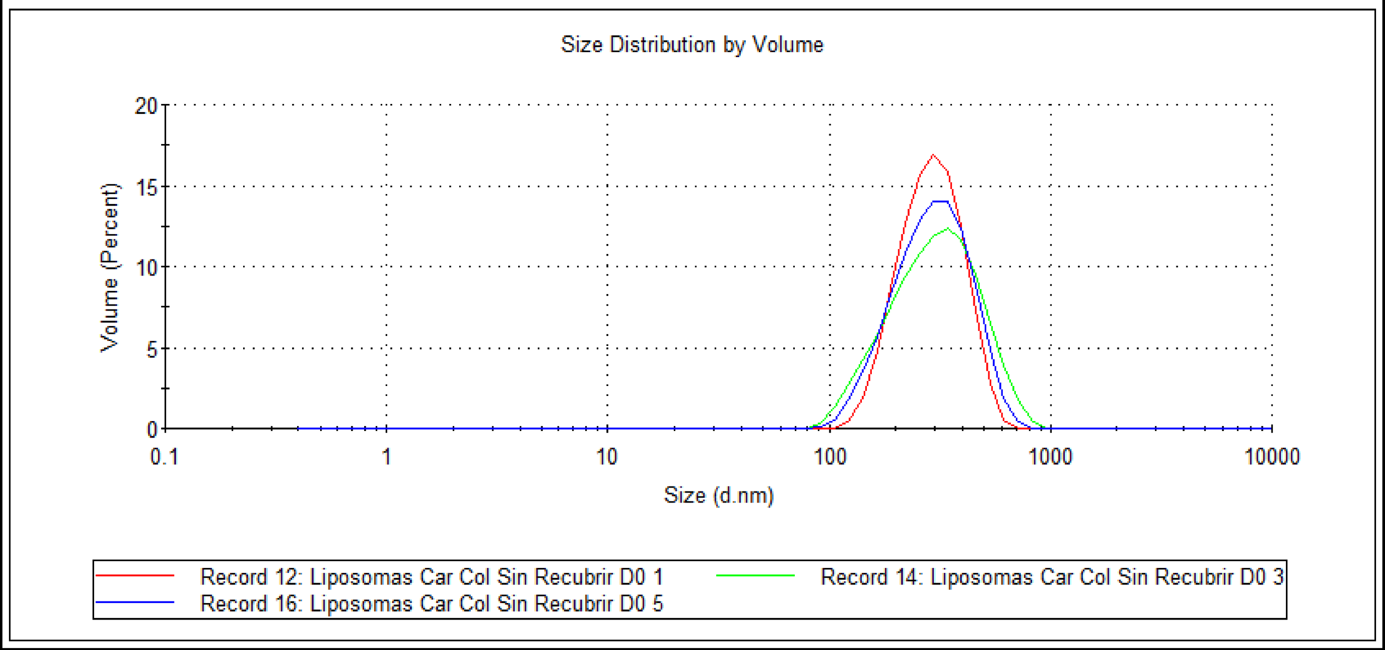

(E) Ch-HNPs

Temperature (°C):	25,0	Duration Used (s):	45
Count Rate (kcps):	182,0	Measurement Position (mm):	4,65
Cell Description:	Disposable sizing cuvette	Attenuator:	7

Correlogram Report

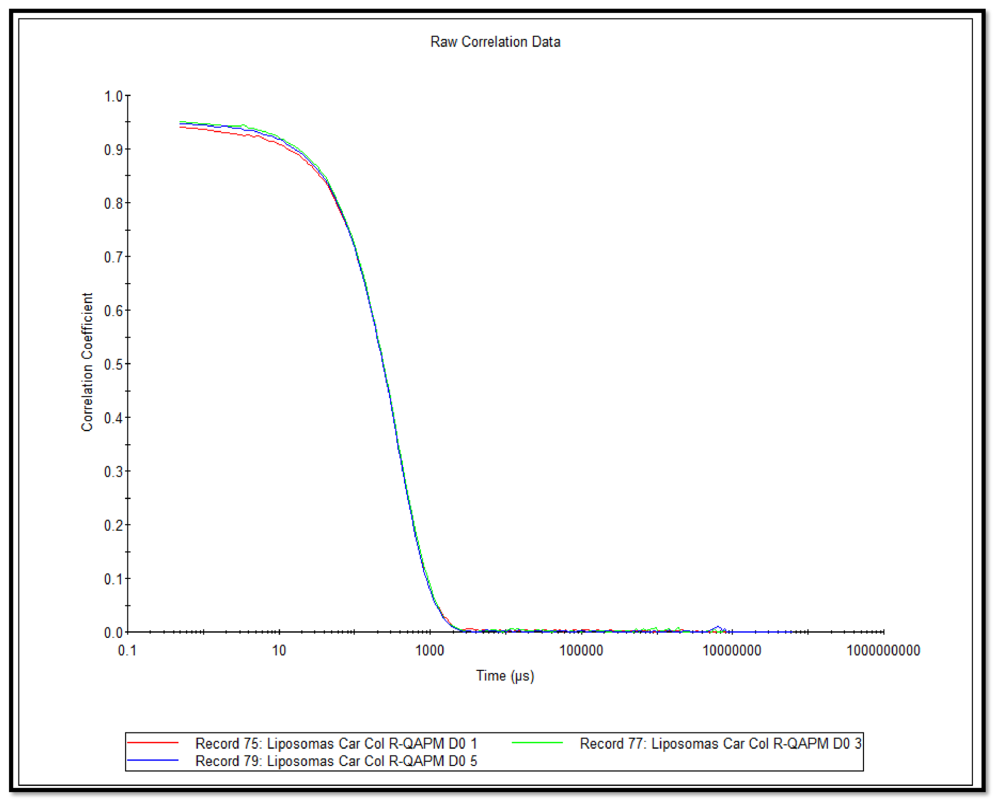

Size Distribution Report by Number

			Size (d.nm):	% Number:	St Dev (d.nm):
Z-Average (d.nm):	247,3	Peak 1:	217,3	100,0	81,09
PdI:	0,138	Peak 2:	0,000	0,0	0,000
Intercept:	0,973	Peak 3:	0,000	0,0	0,000
Result quality :	Good				

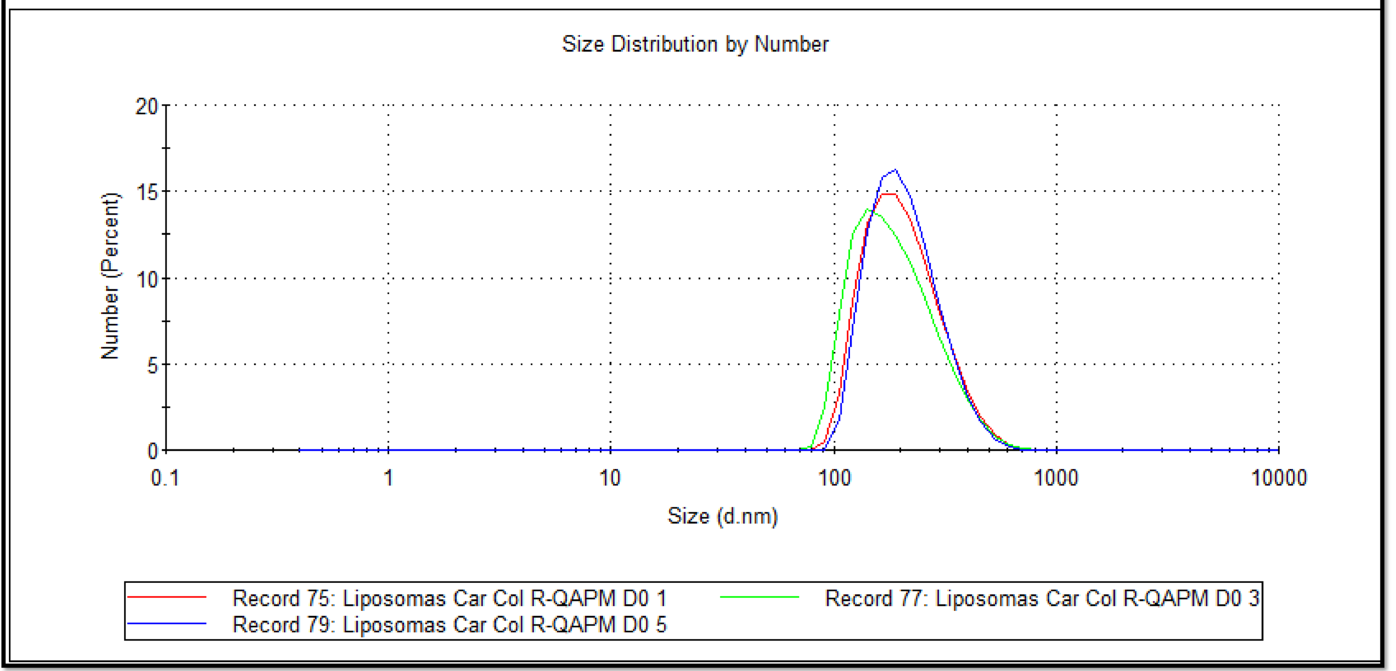

Size Distribution Report by intensity

			Size (d.nm):	% Intensity:	St Dev (d.nm):
Z-Average (d.nm):	247,3	Peak 1:	284,8	100,0	98,48
PdI:	0,138	Peak 2:	0,000	0,0	0,000
Intercept:	0,973	Peak 3:	0,000	0,0	0,000
Result quality :	Good				

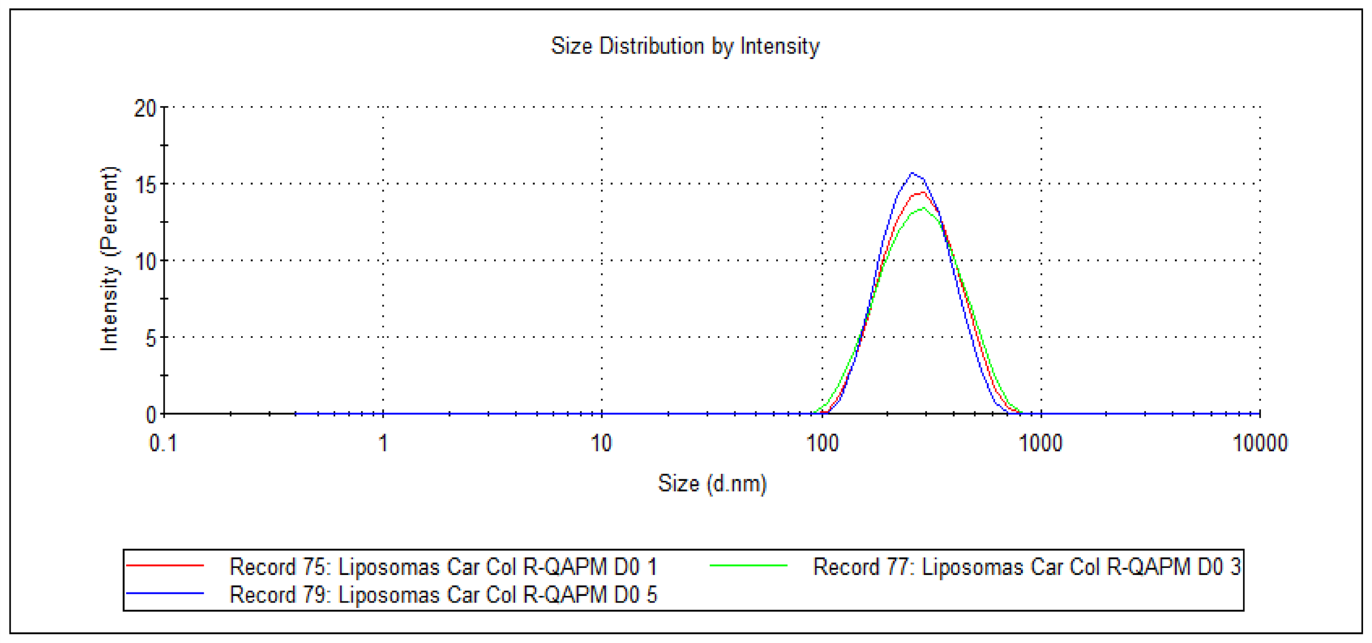

Size Distribution Report by Volume

			Size (d.nm):	% Volume:	St Dev (d.n...
Z-Average (d.nm):	247,3	Peak 1:	313,6	100,0	112,4
PdI:	0,138	Peak 2:	0,000	0,0	0,000
Intercept:	0,973	Peak 3:	0,000	0,0	0,000
Result quality :	Good				

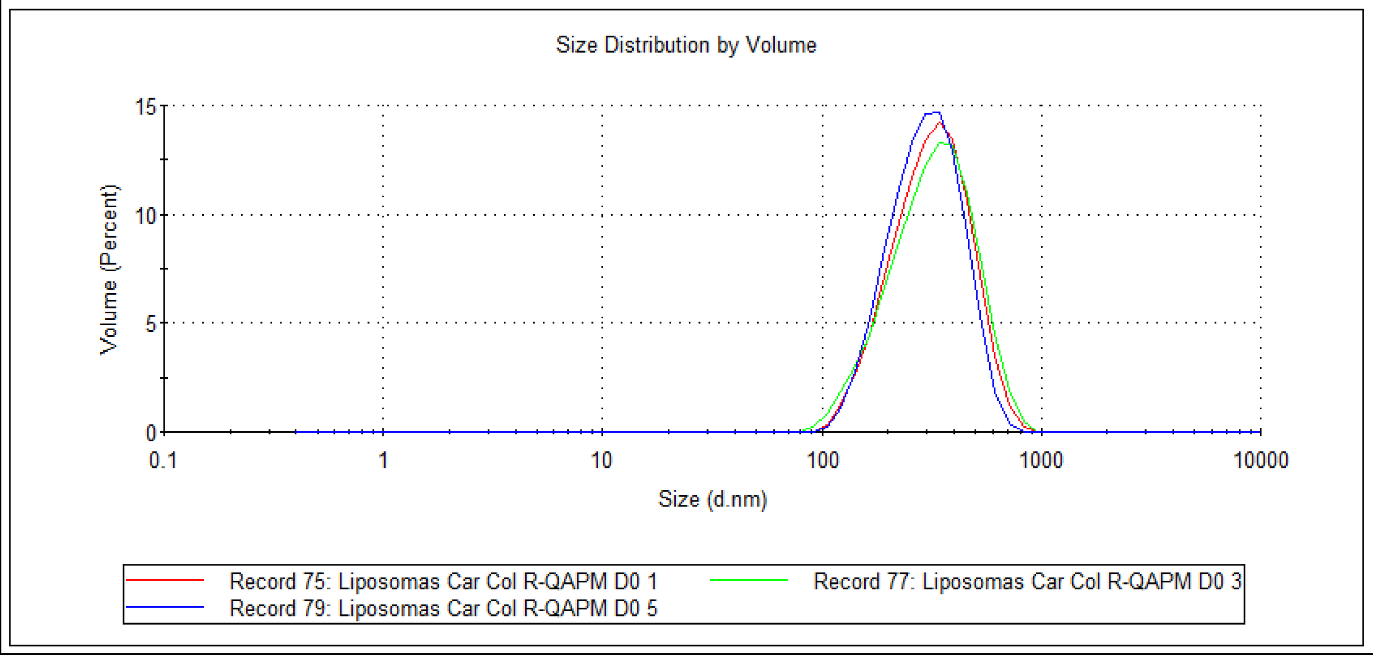

Zeta potential

(A) Colistin aqueous dispersion (0.29 mM)

Temperature (°C):	25,0	Zeta Runs:	10
Count Rate (kcps):	63,5	Measurement Position (mm):	2,00
Cell Description:	Clear disposable zeta cell	Attenuator:	10

Zeta Potential Report

			Mean (mV)	Area (%)	St Dev (mV)
Zeta Potential (mV):	67,5	Peak 1:	67,5	100,0	3,90
Zeta Deviation (mV):	3,90	Peak 2:	0,00	0,0	0,00
Conductivity (mS/cm):	0,239	Peak 3:	0,00	0,0	0,00

Result quality : Good

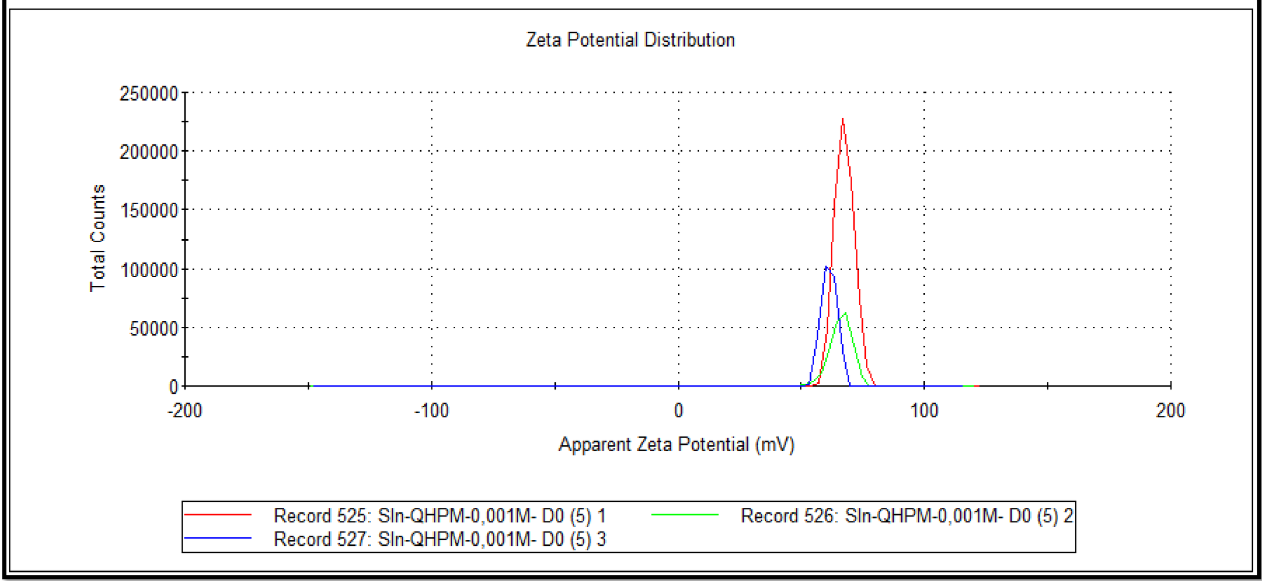

Phase Plot

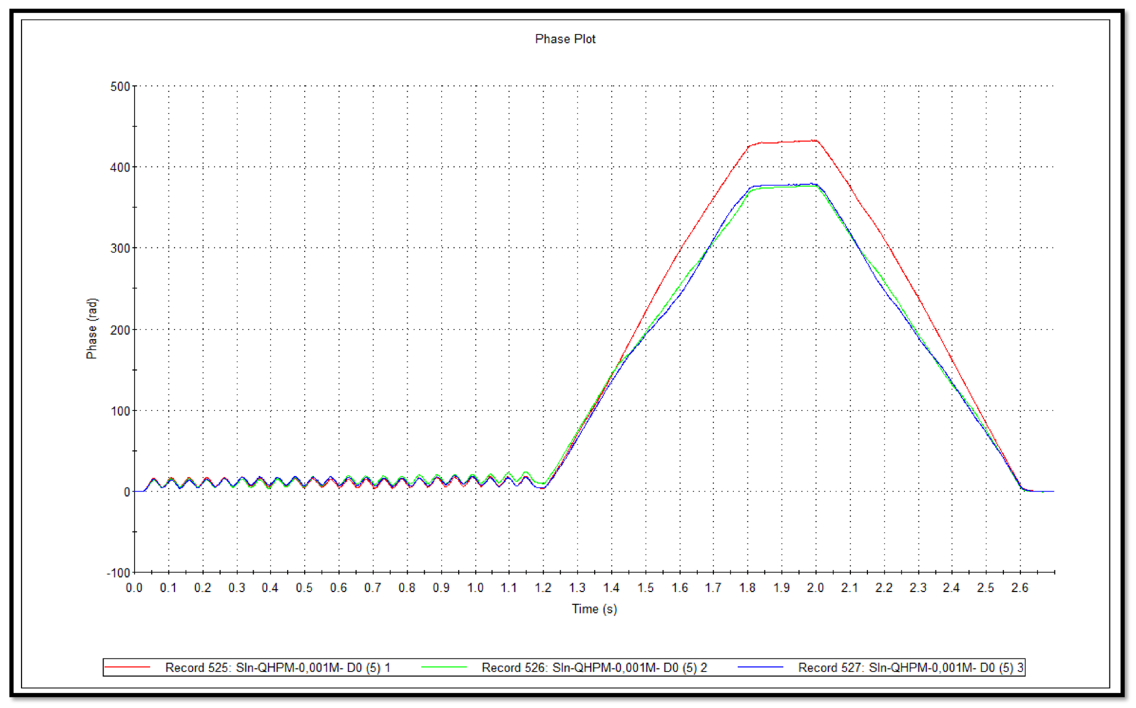

(B) Phospholipon ® aqueous dispersion (0.06 mM)

Temperature (°C):	25,0	Zeta Runs:	14
Count Rate (kcps):	74,8	Measurement Position (mm):	2,00
Cell Description:	Clear disposable zeta cell	Attenuator:	6

Zeta Potential Report

			Mean (mV)	Area (%)	St Dev (mV)
Zeta Potential (mV):	-7,16	Peak 1:	-7,16	100,0	8,06
Zeta Deviation (mV):	8,06	Peak 2:	0,00	0,0	0,00
Conductivity (mS/cm):	2,06	Peak 3:	0,00	0,0	0,00

Result quality : Good

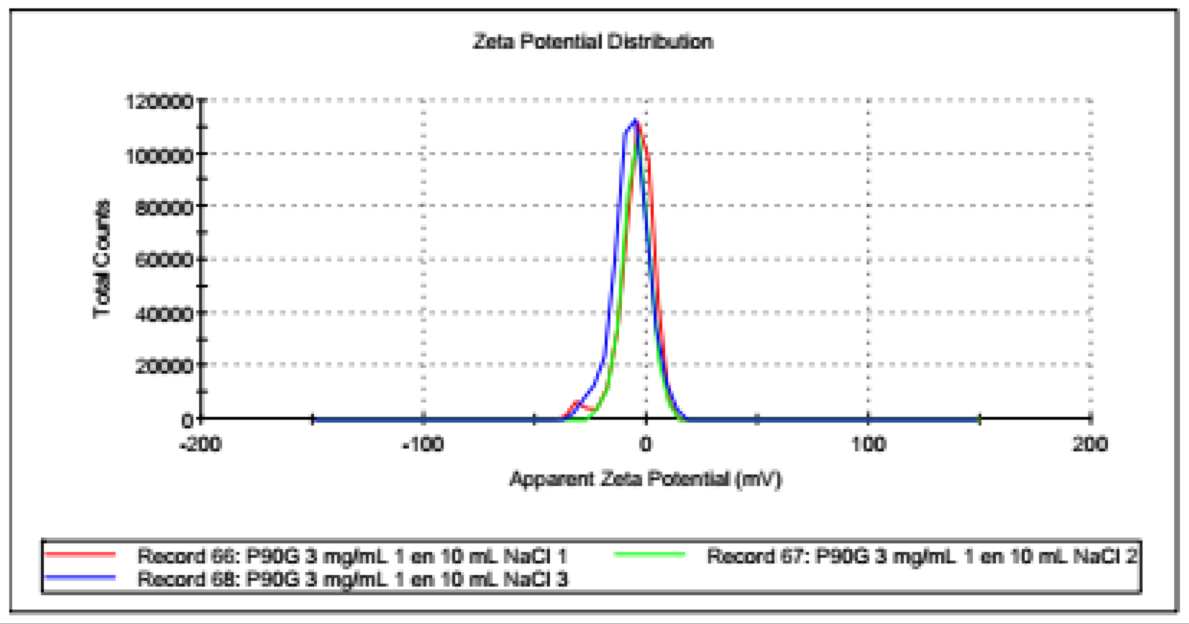

Phase Plot

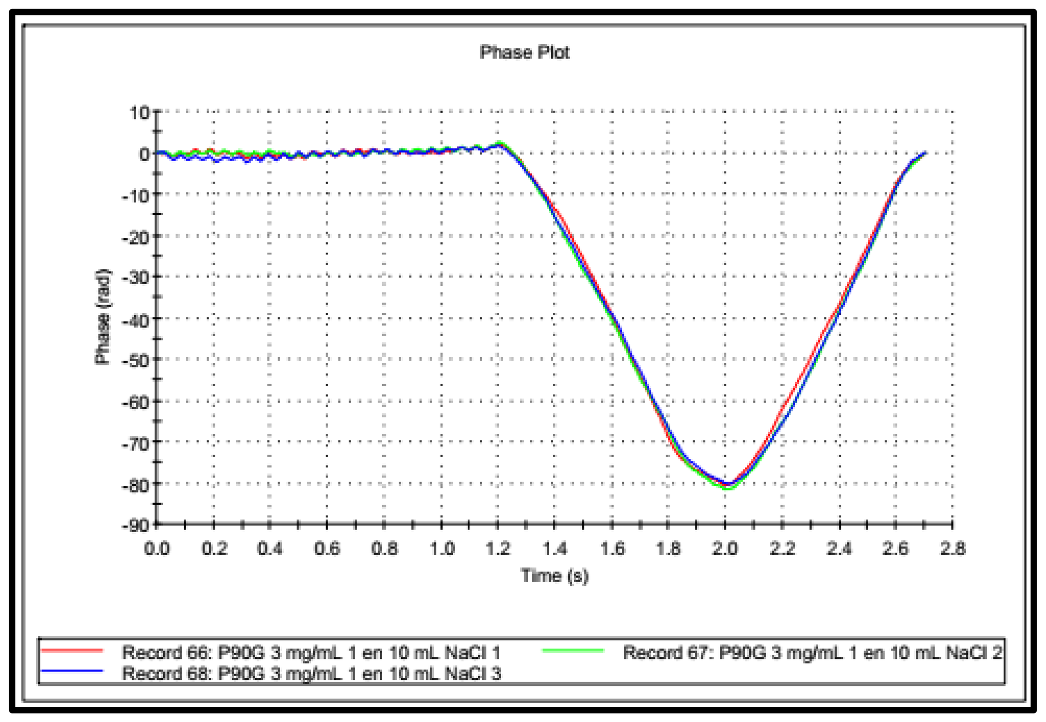

(C) Chitosan aqueous dispersion (1.0 mM)

Temperature (°C):	25,0	Zeta Runs:	8
Count Rate (kcps):	232,2	Measurement Position (mm):	2,00
Cell Description:	Clear disposable zeta cell	Attenuator:	7

Zeta Potential Report

			Mean (mV)	Area (%)	St Dev (mV)
Zeta Potential (mV):	11,2	Peak 1:	11,2	100,0	3,36
Zeta Deviation (mV):	3,36	Peak 2:	0,00	0,0	0,00
Conductivity (mS/cm):	0,155	Peak 3:	0,00	0,0	0,00

Result quality : Good

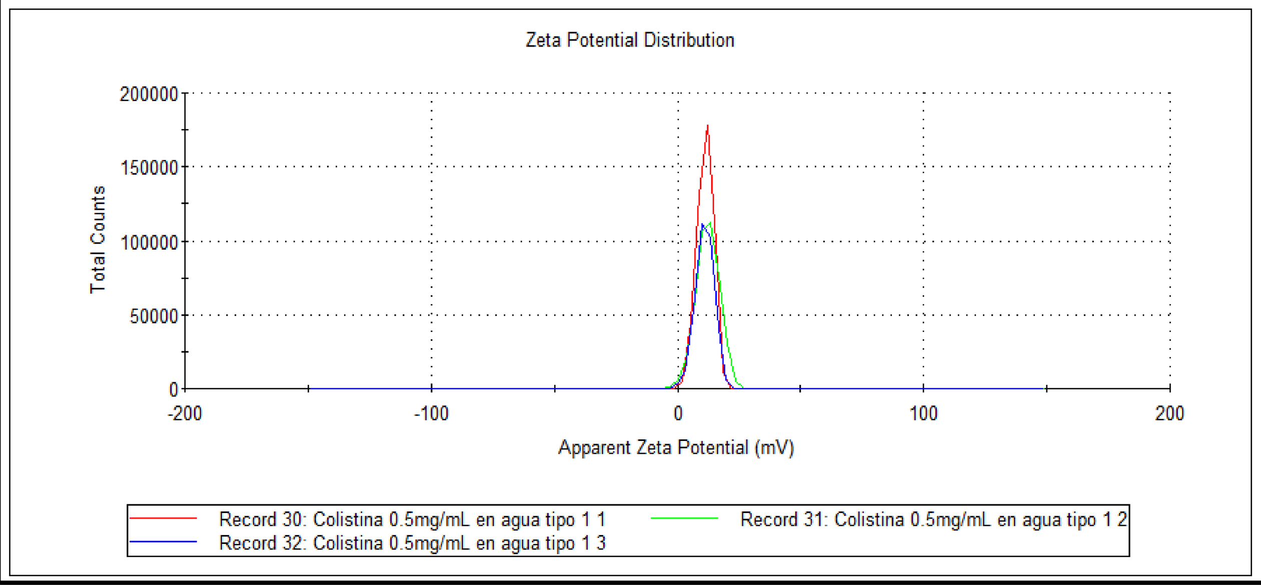

Phase Plot

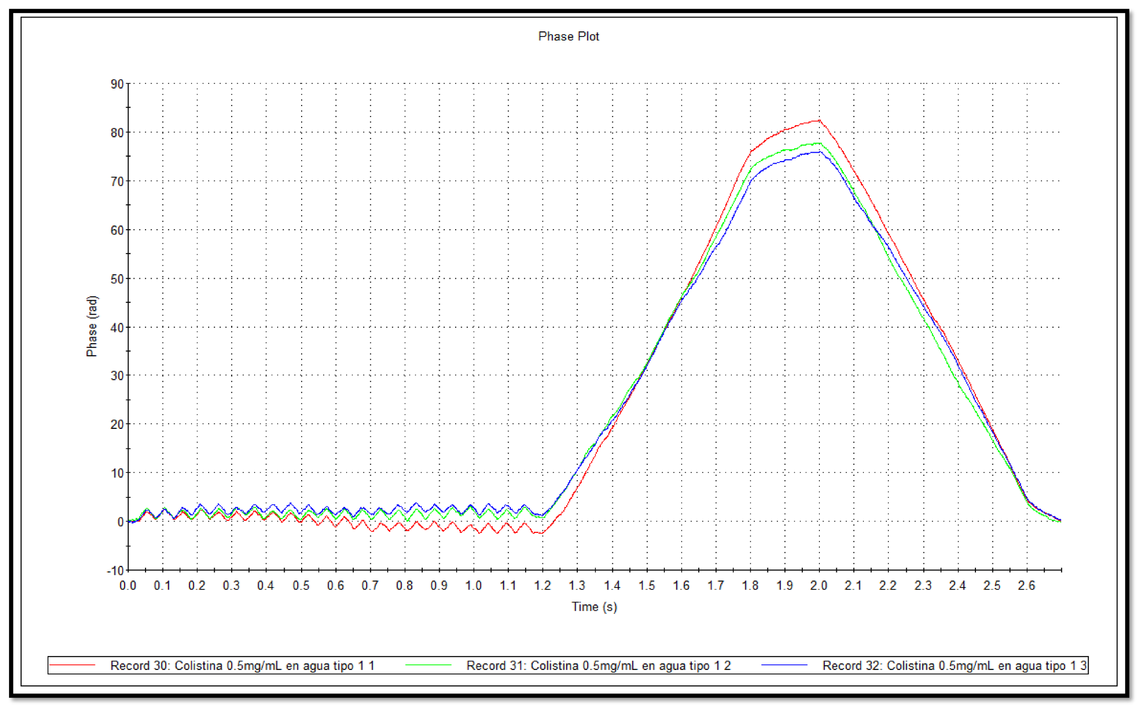

(D) U-HNPs

Temperature (°C):	25,0	Zeta Runs:	8
Count Rate (kcps):	331,5	Measurement Position (mm):	2,00
Cell Description:	Clear disposable zeta cell	Attenuator:	5

Zeta Potential Report

			Mean (mV)	Area (%)	St Dev (mV)
Zeta Potential (mV):	17,3	Peak 1:	17,3	100,0	4,67
Zeta Deviation (mV):	4,67	Peak 2:	0,00	0,0	0,00
Conductivity (mS/cm):	0,185	Peak 3:	0,00	0,0	0,00

Result quality : Good

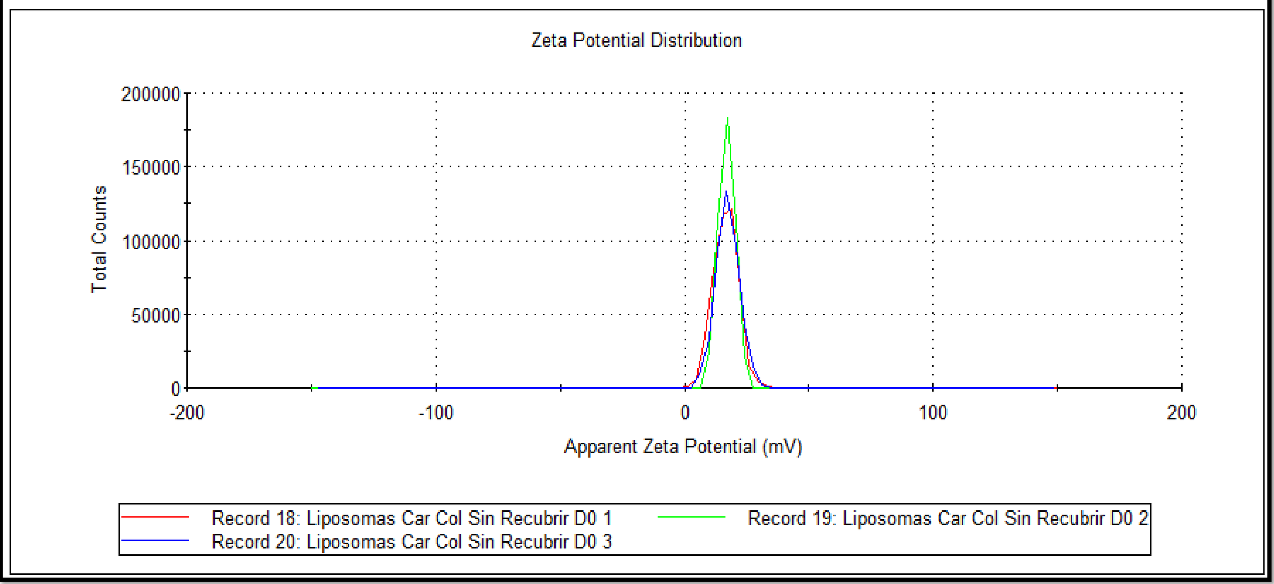

Phase Plot

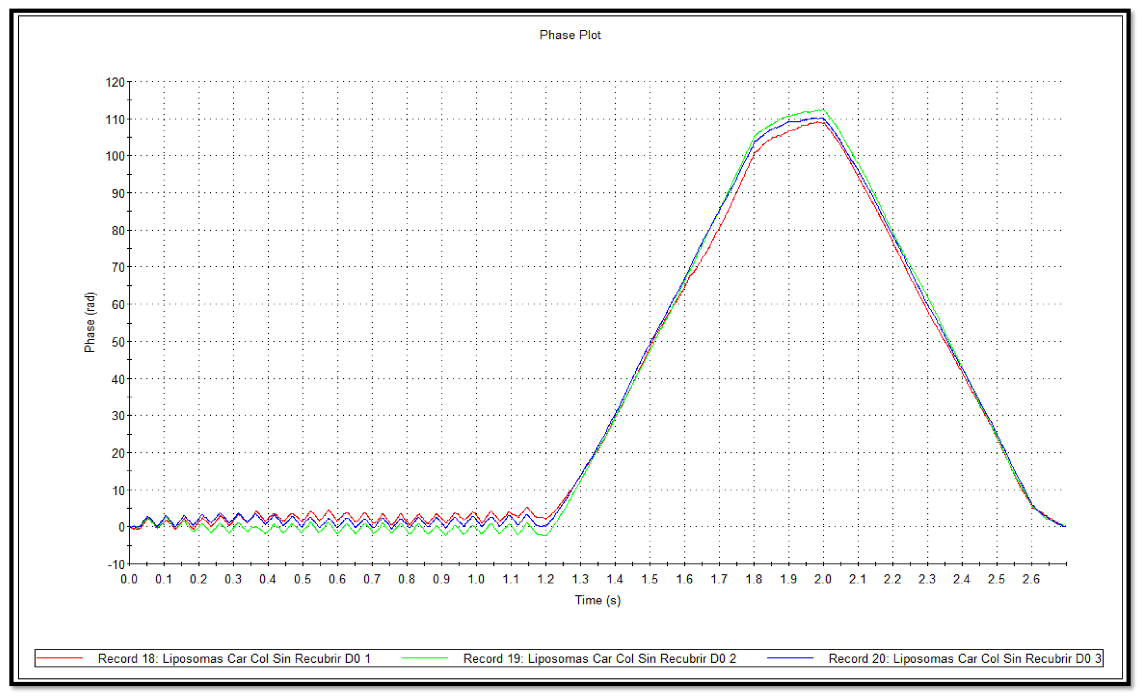

(E) Ch-HNPs

Temperature (°C):	25,0	Zeta Runs:	8
Count Rate (kcps):	114,7	Measurement Position (mm):	2,00
Cell Description:	Clear disposable zeta cell	Attenuator:	5

Zeta Potential Report

			Mean (mV)	Area (%)	St Dev (mV)
Zeta Potential (mV):	21,2	Peak 1:	21,2	100,0	3,75
Zeta Deviation (mV):	3,75	Peak 2:	0,00	0,0	0,00
Conductivity (mS/cm):	0,195	Peak 3:	0,00	0,0	0,00

Result quality : Good

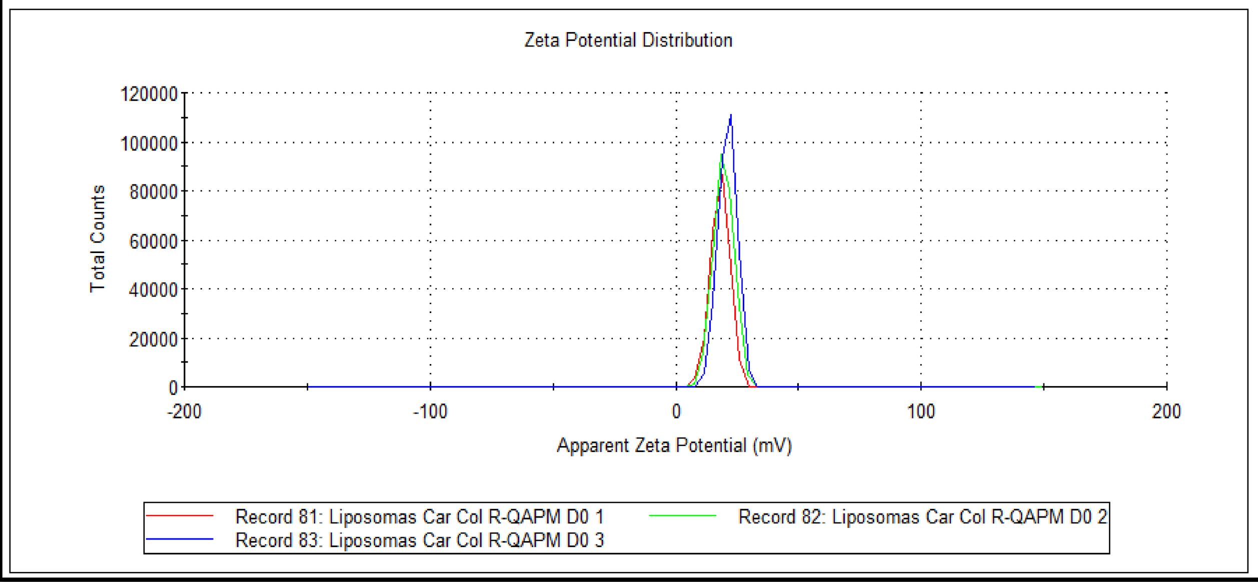

Phase Plot

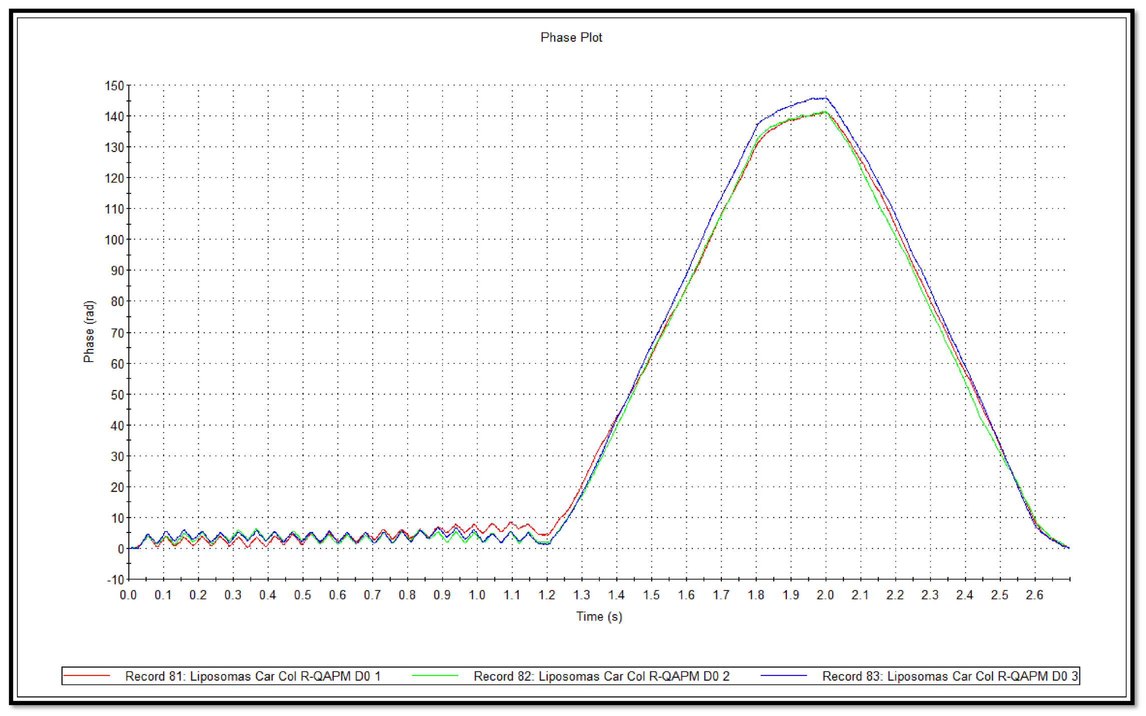

Supplement: Supplementary file 1 [file pharmaceutics-17-00182-s001.zip › pharmaceutics-3405972-supplementary.pdf]
